# Promising prospects of nanopore sequencing for algal hologenomics and structural variation discovery

**DOI:** 10.1186/s12864-019-6248-2

**Published:** 2019-11-13

**Authors:** Thomas Sauvage, William E. Schmidt, Hwan Su Yoon, Valerie J. Paul, Suzanne Fredericq

**Affiliations:** 1 Smithsonian Marine Station, Florida, USA; 2 Biology Department, University of Louisiana at Lafayette, Louisiana, USA; 3 Department of Biological Sciences, Sungkyunkwan University, Suwon, Korea

**Keywords:** Heteroplasmy, Homing endonuclease, Retrohoming, MinION, *Caulerpa*, Ulvophyceae, Holobiont, Rhodospirillaceae, Symbiont, Microbiome

## Abstract

**Background:**

The MinION Access Program (MAP, 2014–2016) allowed selected users to test the prospects of long nanopore reads for diverse organisms and applications through the rapid development of improving chemistries. In 2014, faced with a fragmented Illumina assembly for the chloroplast genome of the green algal holobiont *Caulerpa ashmeadii*, we applied to the MAP to test the prospects of nanopore reads to investigate such intricacies, as well as further explore the hologenome of this species with native and hybrid approaches.

**Results:**

The chloroplast genome could only be resolved as a circular molecule in nanopore assemblies, which also revealed structural variants (i.e. chloroplast polymorphism or heteroplasmy). Signal and Illumina polishing of nanopore-assembled organelle genomes (chloroplast and mitochondrion) reflected the importance of coverage on final quality and current limitations. In hybrid assembly, our modest nanopore data sets showed encouraging results to improve assembly length, contiguity, repeat content, and binning of the larger nuclear and bacterial genomes. Profiling of the holobiont with nanopore or Illumina data unveiled a dominant Rhodospirillaceae (Alphaproteobacteria) species among six putative endosymbionts. While very fragmented, the cumulative hybrid assembly length of *C. ashmeadii*’s nuclear genome reached 24.4 Mbp, including 2.1 Mbp in repeat, ranging closely with GenomeScope’s estimate (> 26.3 Mbp, including 4.8 Mbp in repeat).

**Conclusion:**

Our findings relying on a very modest number of nanopore R9 reads as compared to current output with newer chemistries demonstrate the promising prospects of the technology for the assembly and profiling of an algal hologenome and resolution of structural variation. The discovery of polymorphic ‘chlorotypes’ in *C. ashmeadii*, most likely mediated by homing endonucleases and/or retrohoming by reverse transcriptases, represents the first report of chloroplast heteroplasmy in the siphonous green algae. Improving contiguity of *C. ashmeadii*’s nuclear and bacterial genomes will require deeper nanopore sequencing to greatly increase the coverage of these larger genomic compartments.

## Background

Single molecule sequencing, often referred to as third generation sequencing [1, 2] (e.g. Pacific Bioscience or Oxford Nanopore Technologies), allows the sequencing of long DNA molecules spanning complex genomic regions. In contrast, short read second generation sequencers (e.g. Illumina or Ion semiconductor technologies) can only attempt the bioinformatic reconstruction of such regions. Among the above, Oxford Nanopore Technologies (ONT) is a relatively recent company whose sequencers enable the routine decoding of kilobase single molecules. ONT’s first sequencer, named the MinION, became commercially available in 2015 following an early phase of testing by selected users within the MinION Access Program (MAP). The MAP included the diminutive MinION device, two flow cells and a library preparation kit of choice renewed to users for each new chemistry release.

All Oxford nanopore platforms function on the basis of the same principle (and nanopore chemistries) for DNA sequencing, in which a single-stranded DNA (ssDNA) unfolding from a double stranded DNA (dsDNA) molecule threads through a nanopore embedded in a membrane to which voltage is applied. The translocation of the ssDNA through the nanopore results in a drop in the ion current (raw electrical current aggregated into ‘events’), which varies for different sets of bases (i.e. translocation occurs in a sliding window fashion, previously 5-mers, but now 6-mers [3]). The recorded events are then basecalled into a DNA sequence by dedicated software (locally or in the cloud), and following genome assembly, the raw signal can be accessed to polish contigs, call variants or detect methylation (e.g. with Nanopolish [4, 5]). Two main classes of nanopore reads can be generated - they include 1D reads (D for Dimension or Direction) and higher quality consensus reads formed by reading both the template and complement strands of a given dsDNA molecule. However, to produce such consensus reads, different adapters are required so that the template and complement can thread successively through the nanopore. Early on, this was achieved with a ligated hairpin that physically linked complementary strands into a 2D molecule (up until 2016), while in the latest chemistry (R9.5, released in 2017), linear adapters exhibiting molecular affinity encourage the complementary strand to immediately follow the template strand in the nanopore without direct physical linking, thus the renaming of 2D to 1D^2^.

Several variables, both intrinsic and extrinsic, affect the throughput of MinION flow cells. The former relates to the number of viable nanopores present on the flow cell and the speed of sequencing (i.e. threading), while the latter relates to DNA quality and fragment size distribution of the sequencing library. MinION flow cells are built with up to 2048 nanopores (grouped by 4 in channels) but only a maximum of 512 can thread molecules simultaneously (i.e. 1 per channel), with the best nanopores being partitioned at run start and switched on over time according to a so-called ‘mux scan’ [3]. Thus, because the population of nanopores on a flow cell is finite, the actual number of viable nanopores at run start (i.e. flow cell quality) and the sequencing speed have a major impact on throughput. Since the MinION early days (2014), the translocation of ssDNA through nanopores has increased by 15-fold; indeed, the first chemistry (R6) threaded ssDNA at 30 base pair/second (i.e. bps), jumping to 70 bps in 2015 (R7/R7.3) and 250 bps in the summer of 2016 (R9) to reach 450 bps in the Fall of 2016/Spring 2017 (R9.4/9.5), with ONT reporting that 1000 bps may be possible on the current electronics.

**Fig. 1:**
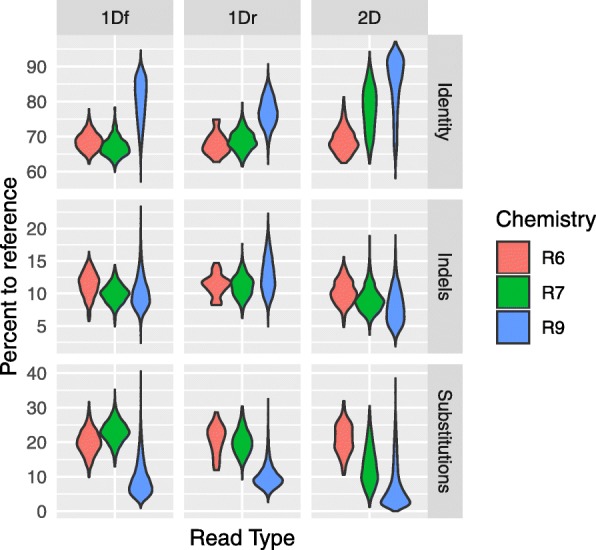
Progression of nanopore read quality. Violin plot depicting improvement of raw nanopore reads quality from chemistry R6, R7/7.3 to R9. Quality is reported in terms of percentage identity, indels and substitutions measured by BLASTn against the final curated chloroplast genome. Raw reads were basecalled in the cloud with the default version of Metrichor available at time of sequencing

These iterations in chemistry were also accompanied by an increase in read quality. From 2014 to 2016, nanopore users saw a rapid progression of reads’ percent identity (up to ~ 20%), thanks to decreased indels (i.e. insertions/deletions) and decreased substitutions errors (i.e. mismatches) ([6, 7]). As reported by others (see Fig. 1g in [8]), we observed that the main improvement between the early chemistries R6 and R7.3 was primarily seen in consensus 2D reads (i.e. little to no improvement in 1D reads), while moving from R7/7.3 to R9, the quality of both 1D and 2D reads ameliorated notably (Fig. 1). With the release of R9 chemistry, the difference in percent identity between 1D and 2D reads also greatly narrowed (~ 5%, [9]) with many reads actually overlapping in quality (80–90% identity to reference, Fig. 1). ONT claims that with current chemistries, most 1D and consensus reads 1D^2^ reach 90 and 95% identity to reference, respectively.

**Fig. 2:**
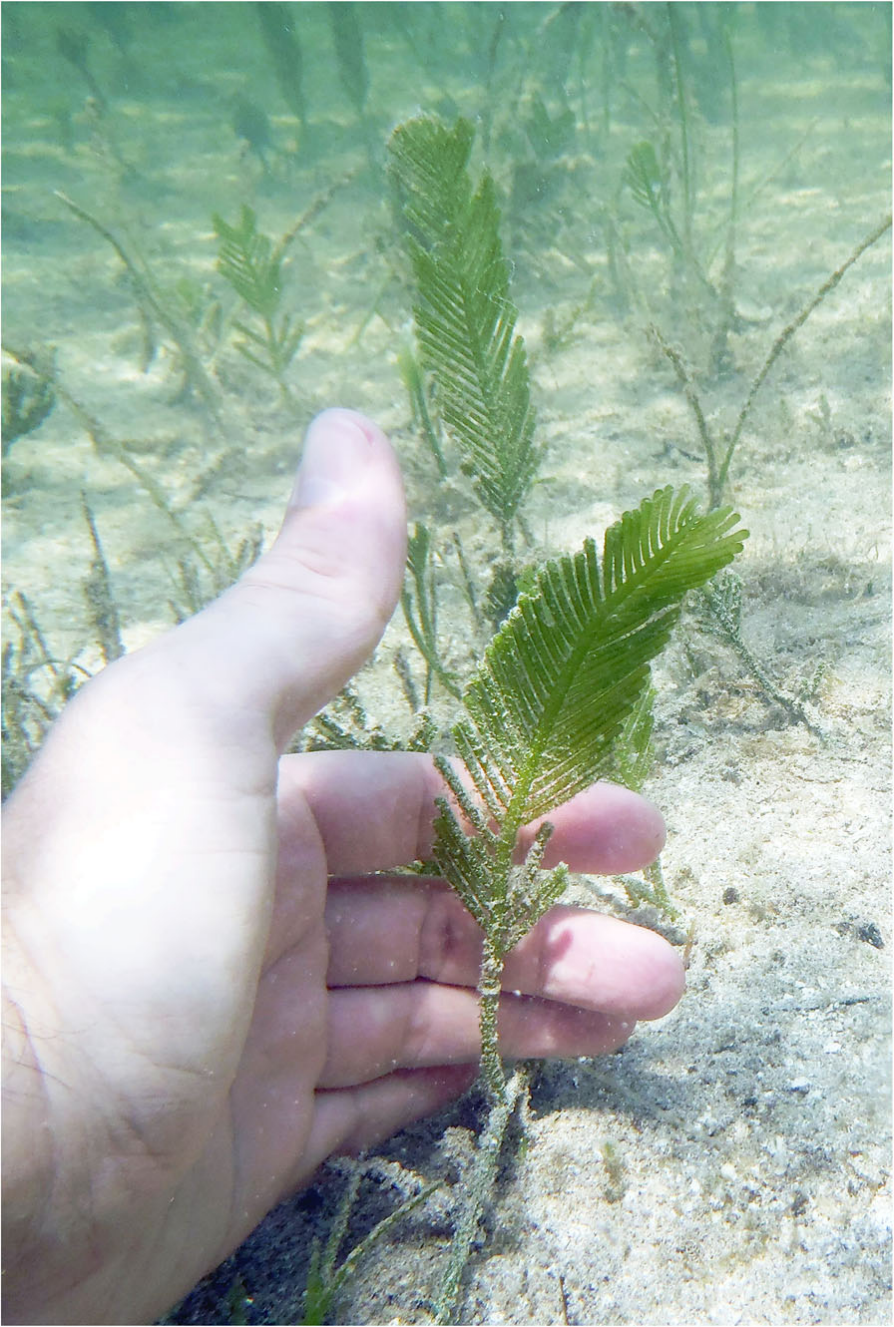
*Caulerpa ashmeadii* in the field. Typical habit of *Caulerpa ashmeadii* exhibiting large distichous fronds growing erected from stolons anchored by their rhizoids in soft bottom habitats

In 2014, faced with a fragmented Illumina assembly for the chloroplast genome of the green algal holobiont *Caulerpa ashmeadii* Harvey 1858 (Bryopsidales, Ulvophyceae, Chlorophyta) (Fig. 2), we applied to the MinION Access Program (MAP) to test the prospects of ONT’s long reads to resolve the issue, and further explore the metagenome (i.e. hologenome) of this species. Indeed, *Caulerpa* species are siphonous holobionts that develop multinucleated giant cells (or coenocyte) devoid of compartmentalization [10], in which the host’s nuclei, chloroplasts, mitochondria and prokaryotic endosymbionts (i.e. endophytic, e.g. [11]) are present in a common cytoplasmic space, and may function collectively as a hologenome [12, 13]. *Caulerpa* spp. are popular in the aquarium trade [14] and infamous alien species [15, 16], emerging as model species to study algal hologenomes [17].

In the present study, we relate our experience with the MAP for the sequencing of DNA extracted from the fronds of *C. ashmeadii*. Based on modest nanopore 1D and 2D data sets collected during our last testing of the technology with R9 chemistry (August 2016), we report on simple mitigation strategies to improve read size distribution and results of assemblies and genome polishing performed in native and hybrid frameworks. Relying on hybrid assemblies, we also identify and draw profiles of associated bacterial communities and provide an outlook of nuclear genome size and repeat content. Finally, to gain perspective on our nanopore runs and resequencing prospects, we review MinION flow cell output published by users for R9 and R9.4.

## Results

### Nanopore libraries

**Fig. 3:**
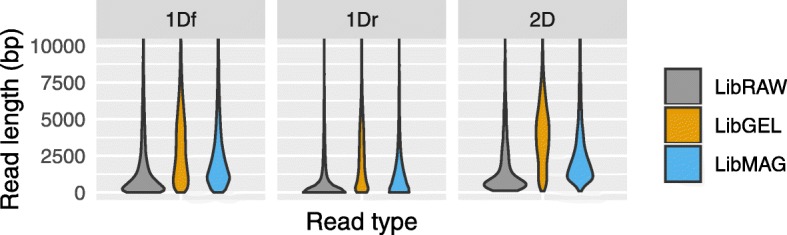
Read length distribution. Violin plot depicting read length improvement following decontamination of low molecular weight fragment (LMW). Lib_RAW_: original DNA extract, Lib_GEL_: Gel excised HMW DNA, and Lib_MAG_: HMW DNA selected via 0.4X magnetic bead wash. Data is shown for 1Df, 1Dr and 2D reads. Note the broader shoulder and distribution of Lib_GEL_ for sequence > 2500 bp. (See Additional file 2: Table S1 for a numerical summary and Additional file 3: Figure S1 for density plots)

Among the two R9 flow cell tested, the first one was run with raw genomic DNA (Library Lib_RAW_) and led to poor results because of the presence of excessive low molecular weight (LMW) fragments (Fig. 3), few active pore numbers, and probably inadequate DNA concentration of the sequencing library (inaccuracy of NanoDrop measurements, see methods and Additional file 1). Steps taken to mitigate these issues on a second R9 flow cell (i.e. by increasing library concentration and testing two LMW decontamination strategies), led to much improved read numbers (see Table 1) and distribution of read sizes (Fig. 3, Additional file 3: Figure S1, Additional file 2: Table S1). Excision of HMW on gel (Library Lib_GEL_) seemed particularly efficient to deplete LMW, as seen from the broader density trace of read length distribution above 2500 bp for all read types (template 1Df, complement 1Dr and consensus 2D, Fig. 3, Additional file 3: Figure S1). Likewise, performing a single 0.4X magnetic wash (Library Lib_MAG_) greatly decreased LMW fragments, grossly doubling median read sizes (Additional file 2: Table S1). Lib_GEL_ and Lib_MAG_ read counts were similar (each ~ 90 k), but Lib_GEL_ logically translated in a larger base pair output (Table 1) considering its larger fragment size distribution. In general, and regardless of library, 1Df and 2D reads exhibited larger lengths than 1Dr reads (Fig. 3). For read counts, 1Df reads were generally twice more abundant than 1Dr reads (48% more) and five times more abundant than consensus 2D reads (Table 1). Overall, after combining the three nanopore libraries and filtering out reads < 1000 bp, a total of > 143 k 1Df reads (> 540 Mbp) and > 37 k 2D reads (> 120 Mbp) were available for assembly (1Dr reads were not used for assembly, see further details in methods).

**Table 1:** Flow cell output

Flow Cell: Library	1Df	1Dr	1Df + 1Dr	2D
FAB29720: Lib_RAW_	11,784	6622	18,406	1486
	22.9 Mbp	10.2 Mbp	33.1 Mbp	2.6 Mbp
FAB38981: Lib_GEL_	90,189	47,119	137,308	18,557
	296.4 Mbp	114.4 Mbp	410.8 Mbp	73.2 Mbp
Lib_MAG_	91,503	40,148	131,651	17,843
	245.6 Mbp	66.5 Mbp	312.1 Mbp	47.9 Mbp
Total	181,692	87,267	268,959	36,400
	542 Mbp	180.9 Mbp	722.9 Mbp	121.1 Mbp
Grand Total	193,476	93,889	287,365	37,886
	564.9 Mbp	191.1 Mbp	756 Mbp	123.7 Mbp
Filtered Total	143,797	51,687	195,484	32,177
	540.1 Mbp	175.8 Mbp	715.9 Mbp	120.1 Mbp

Number of reads and cumulative length generated per read class, library and flow cell. Filtered 1Df and 2D reads (> 1000 bp) were used for nanopore or hybrid assembly

### Organelle assemblies

**Fig. 4:**
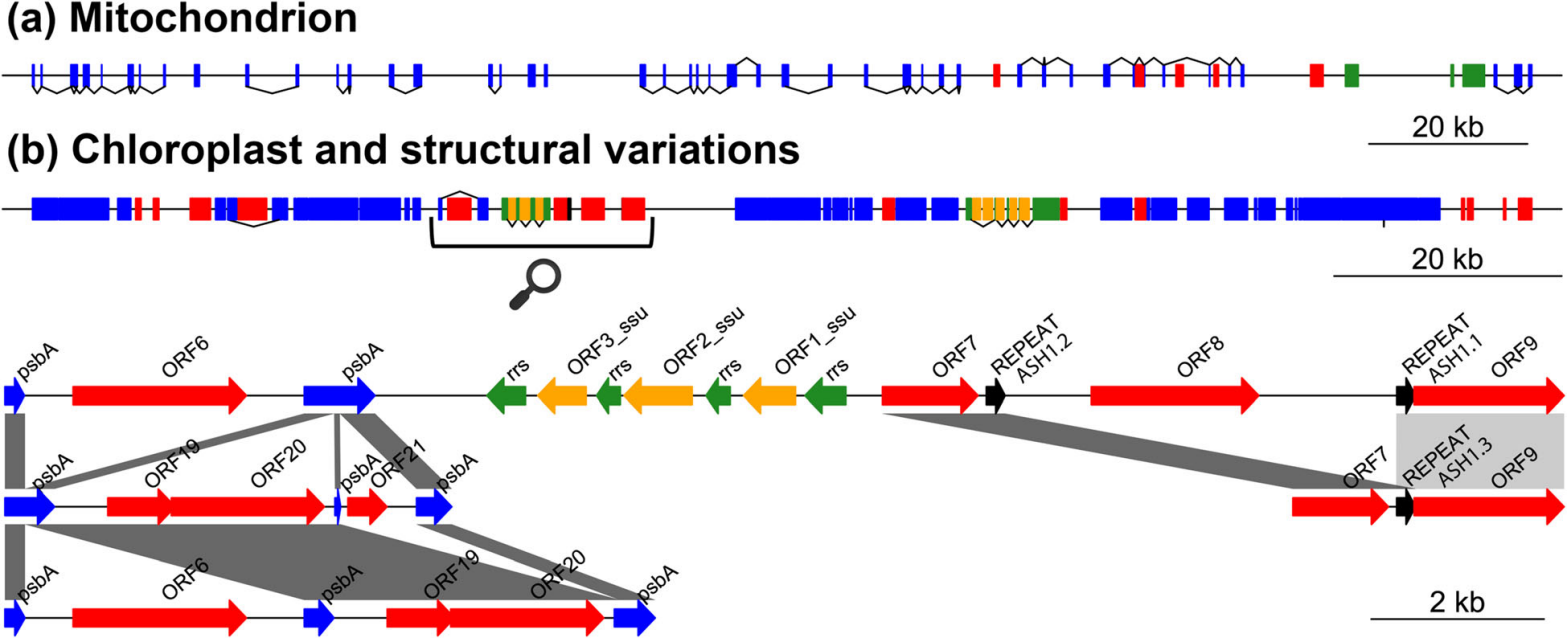
Organellar genomes and structural variation. Overview of organellar genome content and structural variation (intronic ORF presence/absence) in *Caulerpa ashmeadii*. **a** Mitochondrial genome (197 Kbp, linearized). **b** Chloroplast genome (135 Kbp, linearized) with region of discovered structural variation and interspersed repeats shown with the bracket and cartoon magnifier. Colored blocks represent protein-coding genes and cassettes (in blue), free-standing ORFs (red), ribosomal DNA (rDNA) and associated ORFs (green and orange respectively). Gene blocks connected by lines represent exons of a same gene. Lines indicate sense transcription when placed above the genome, and antisense transcription when placed below. Note structural variation of the *psb*A intron and interspersed repeats ASH1.1/ASH1.2 recombining in repeat ASH1.3. Note also the large number of introns in the mitochondrial genome and lower overall gene content and compactness. (see Additional file 5: Figure S3 and Additional file 6: Figure S4 for a circular view of the organellar genomes further detailing gene content)

While sorting the bacterial and nuclear genomes required binning, identifying the chloroplast (CP) and mitochondrion (MT) genomes from assembly files was straightforward via BLASTn. All assemblies revealed a large CP contig/scaffold and multiple shorter ones corresponding to structural variants (SVs) (see green data points, Additional file 4: Figure S2, and later section). However, the CP genome could only be circularized in the nanopore assemblies (both 1Df and 2D). In contrast, the largest CP scaffold in the Illumina assembly could not be circularized because it was only partial (i.e. fragmented), while in the hybrid assemblies, the ‘stitching’ of SVs on the scaffold’s extremities, prevented their circularization. The mitochondrial genome was in comparison straightforward to assemble (see red data points, Additional file 4: Figure S2) since a single contig/scaffold was present in each assembly file, except in the 2D nanopore assembly because of insufficient sequencing depth (~10X, see Table 2). The MT genome showed much lower gene density and numerous introns as compared to the CP (Fig. 4a, Additional file 5: Figure S3 and Additional file 6: Figure S4), and following circularization and curation, the complete CP and MT genomes were 135,722 bp and 197,427 bp, respectively. Overall, the CP and MT genomes accumulated important coverage as compared to the much larger bacterial and nuclear genomes (Table 2), allowing exploration of polishing tools, read class and abundance on genome quality improvement (see corresponding section).

**Table 2:** Read counts, relative abundance and coverage per genomic compartment and dataset

	1Df	2D	Illumina PE
CP	28,530	8157	7,732,624
	29.85%	31.56%	31.28%
	814.7X (± 176.2X)	214.9X (± 42.1X)	7411.2X (± 1177X)
MT	1937	511	742,149
	2.03%	1.98%	3.00%
	41.9X (± 27.2X)	10.5X (± 12.2X)	1084.9X (± 258X)
BACT	49,630	13,786	11,094,869
	51.93%	53.34%	44.89%
	5.6X (± 7.7X)	3.2X (± 3.5X)	160.0X (± 285X)
NU	15,466	3390	5,148,292
	16.18%	13.12%	20.83%
	1.6X (± 1.8X)	1.1X (± 1.1X)	61.2X (± 70X)

Read counts and mean coverage were determined via mapping on the curated chloroplast and mitochondrion genomes or the hybrid 1Df assembly for the nuclear and bacterial compartments. Relative abundance was computed from total read counts for a given dataset. Mapping of nanopore data was conducted with filtered reads (> 1000 bp). Numbers in parenthesis represent coverage standard deviation. Note the low/uneven coverage of the larger nuclear and bacterial genomes. Genome abbreviation as follows, CP = Chloroplast, MT = Mitochondrion, BACT = Bacterial, NU = Nuclear

### Bacterial and nuclear assemblies

Our modest nanopore data sets were insufficient to assemble the bacterial and nuclear genomes as seen from the short cumulative length of the 1Df and 2D assemblies (Table 3). Nuclear reads were less abundant than bacterial ones (Table 2) and resulted in very few nuclear contigs in the 1Df assembly (*n* = 13, Table 3) and no contigs in the 2D assembly (too few reads). However, even at low sampling depth, nanopore assemblies showed a comparatively rapid increase in contiguity over Illumina or hybrid assemblies (see N50 values in Table 3). In a hybrid framework with Spades, the effect of adding nanopore data to improve scaffolding was visible in assembly statistics, although these effects were more conspicuous for the hybrid 1Df assembly, which relied on the most data. Indeed, the hybrid 1Df assembly exhibited an increase of N50 values (i.e. longer and less numerous scaffolds), cumulative assembly length (+ 170 Kbp assembled for BACT and + 141 Kbp for NU, Table 2), and measured repeat content (Table 3 and Additional file 2: Table S2). The hybrid 2D assembly showed some improvement in contiguity as well, but effects on cumulative assembly length and repeat content were inconsistent (Table 3). The total length of our nuclear genome assembly, > 24.3 Mbp, including a repeat length of > 1.9 Mbp, was in very close range with GenomeScope’s estimate of 26.3 Mbp including a repeat length of 4.8 Mbp (heterozygosity of 0.4%, model fit of 99.2%, Additional file 7: Figure S5, Additional file 2: Table S3). Binned bacterial genomes ranged from 2.6 Mbp to 5.5 Mbp (Additional file 2: Table S4). Both nuclear and bacterial genomes remain relatively fragmented (996 and 1345 scaffolds in the hybrid 1Df assembly).

**Table 3:** Nuclear and bacterial assembly statistics

		1Df	2D	Illum. PE	PE+1Df	PE+2D
BACT	N	191	118	1696	1345	1507
	N50	116,795	21,123	35,746	53,173	45,151
	Cumul.	5,461,181	2,185,020	20,445,029	20,615,409	20,573,366
	Repeat	NA	NA	1,282,564	1,376,748	1,251,224
NU	N	13	NA	1192	996	1178
	N50	9441	NA	63,565	79,506	63,762
	Cumul.	107,981	NA	24,338,140	24,479,301	24,335,225
	Repeat	NA	NA	1,938,682	2,093,744	1,904,094

Statistics include the number of Canu contigs/Spades scaffolds (N) and their cumulative assembled length (in bp), computed N50 (in bp), and assembled repeat content (in bp) estimated by RepeatModeler and TRF. BACT = Bacterial, NU=Nuclear, PE = Illumina Paired-Ends, NA = not available

### Binning and profiling

**Fig. 5:**
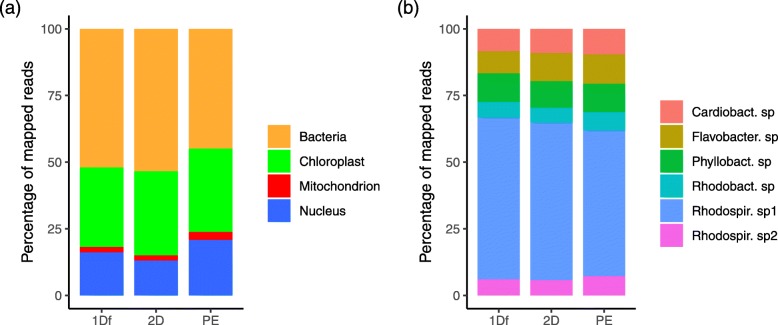
Summary profiles per dataset. Abundance of genomic compartments in the Nanopore (1Df and 2D) and Illumina PE dataset based on read counts determined via mapping on the best hybrid assembly (PE+1Df) (Nuclear and bacterial) or the curated organellar genomes (chloroplast and mitochondrion). **a** Main genomic compartments profiles. **b** Bacterial species profiles. Note the important abundance of the bacterial compartment and of the Rhodospirillaceae sp1 among bacterial species. PE = Illumina Paired-Ends

Binning of hybrid assemblies retrieved the most balanced and largest number of COGs per bins (257 and 255 COGs, vs. 241 for Illumina-only) suggesting better segregation of scaffolds and hologenome completeness. This was particularly true for the hybrid 1Df assembly while the hybrid 2D assembly produced one orphan bin with no COGs (Additional file 8: Figure S6). Performing reciprocal BLASTn across assembly files showed that the binning of nuclear scaffolds was consistent and the delimitation of three bacterial taxa was improved with hybrid scaffolds (i.e. see Additional file 8: Figure S6). Experimental binning of nanopore contigs with MyCC seemed encouraging (e.g. see 1Df, Additional file 9: Figure S7) but recovered very few COGS (1 for 1Df and 8 for 2D), most likely due to protein gene prediction issues caused by frameshifts (i.e. Prodigal in the MyCC workflow [18]). Overall, investigation of the reported COGs with BLASTp for the different bins consistently identified six bacterial taxa; a Cardiobacteriaceae sp., a Flavobacteriaceae sp., a Phyllobacteriaceae sp. (possibly *Hoeflea* sp.), two separate Rhodospirillaceae species, and a Rhodobacteraceae sp. (possibly *Thioclava* sp.) (Additional file 10: Figure S8). Read-wise, the bacterial compartment was the most abundant, nearing ~ 45 to 53% of mapped reads (depending on the dataset), followed by the CP (and its SVs) with 30–32%, the nuclear chromosomes, 13–20% and the mitochondrial genome representing less than 3% (Fig. 5a, Table 2). Within the bacterial compartment, the most abundant species was the Rhodospirillaceae sp. 1 representing ~ 54 to 61% of all bacterial reads (Fig. 5b, Additional file 2: Table S5). Nonetheless, copy-wise (i.e. coverage-wise), the CP and MT genome were the most abundant molecules in the cell (Table 2).

### Organelle polishing and SNPs

Polishing significantly raised the overall quality of the CP and MT genome contigs (Table 4, Additional file 11: Figure S9). Through the different steps of the polishing pipeline and in the case of the 1Df CP genome, the Canu assembled contigs were > 96% identity to reference, with > 3% indels (corresponding to deletion mostly, not shown) and a moderate proportion of substitutions (0.1% or less). Subsequent corrections with Racon decreased indels but increased substitutions (i.e. by correcting some contigs with the wrong bases, Table 4 and Additional file 11: Figure S9). Using Nanopolish for signal-based polishing boosted quality to > 99.6% identity, greatly reducing indels to < 0.4% and substitutions to < 0.01%. Thanks to signal information, Nanopolish also greatly extended homopolymer stretches > 5 bp (Additional file 2: Table S6). Subsequent Illumina-based polishing with Pilon brought quality even higher to > 99.9% identity, mostly filling remaining indels to < 0.1% and with accurate bases (remaining substitutions ~ 0.03%). Interestingly, the quality of the polished 2D CP genome was overall similar (Table 4) despite its lower read coverage (~215X vs. ~815X in 1Df, Table 2). In contrast, the MT genome (i.e. 42X in 1Df) remained lower in quality, tipping at 98.2% with Nanopolish and then 99.7% with Pilon (Table 4, Additional file 11: Figure S9). Interestingly, the nucleotide stretches of protein-encoding regions (i.e. genes) reached slightly higher quality than the entire genome contigs (Table 4, Additional file 11: Figure S9). Finally, comparing Nanopolish and Pilon to detect SNPs for our high coverage organelles determined a single variable position (site 107,088) within the *ycf*4 and *atp*A intergenic span of the CP (Nanopolish found several positions that were not supported by Pilon and vice versa). In the MT, these tools called numerous SNPs in the *atp*1 region, which upon close inspection were revealed in error from the mapping of the CP reads containing the conserved *atp*A gene (~ 70% identity with MT *atp*1, Additional file 12: Figure S10).

**Table 4:** Polished quality of genomes and genes

Assembly	Tool	% Identity	% Indels	% Substitutions
CP 1Df	Canu	96.278 (96.907)	3.615 (3.087)	0.107 (0.006)
	Racon	97.418 (97.777)	2.489 (2.186)	0.093 (0.037)
	Nanopolish	99.622 (99.721)	0.365 (0.265)	0.013 (0.015)
	Pilon	99.929 (100.00)	0.068 (0.000)	0.003 (0.000)
CP 2D	Canu	97.951 (98.344)	2.017 (1.654)	0.032 (0.002)
	Racon	98.184 (98.488)	1.785 (1.496)	0.031 (0.016)
	Nanopolish	99.602 (99.707)	0.403 (0.279)	0.006 (0.015)
	Pilon	99.931 (100.00)	0.066 (0.000)	0.003 (0.000)
MT 1Df	Canu	92.741 (95.767)	6.774 (4.082)	0.485 (0.158)
	Racon	93.133 (95.809)	4.998 (3.131)	1.869 (1.107)
	Nanopolish	98.165 (98.841)	1.190 (0.719)	0.645 (0.445)
	Pilon	99.725 (99.883)	0.183 (0.056)	0.092 (0.061)
MT 2D	–	–	–	–

Quality of nanopore organellar genomes and their protein-encoding genes (between parenthesis) following nanopore assembly (Canu) and polishing with successive tools (Racon, Nanopolish, and Pilon). Note the perfect accuracy of chloroplast genes following Pilon polishing. CP = Chloroplast, MT = Mitochondrion

### Chloroplast SVs

Considering the difficulty of assembling polymorphic regions accurately, even with long read, we cropped 1Df, 2D and hybrid SVs to common stretches for greater confidence while verifying their occurrence at the raw read level. CP SVs consisted of intronic polymorphism in the photosystem II protein D1 gene (*psb*A) and polymorphism of a genomic segment flanked by ~ 200 bp interspersed repeats (annotated as ASH1.1 and ASH1.2) containing an open reading frame (ORF8, Fig. 4b). The gene *psb*A was found in three conformations with variation of the number of exons and exon-intron junctions resulting in ORF presence/absence (ORF6, ORF20 and ORF21/22). The interspersed repeats ASH1.1 and ASH1.2 shared 99% identity and harbored a palindromic site GTTTAAAC possibly acting as a restriction site for an endonuclease to excise the genomic segment containing ORF8, subsequently mediating recombination of the repeat into ASH1.3 (Fig. 4b, Additional file 13: Figure S11).

### Output prospects

**Fig. 6:**
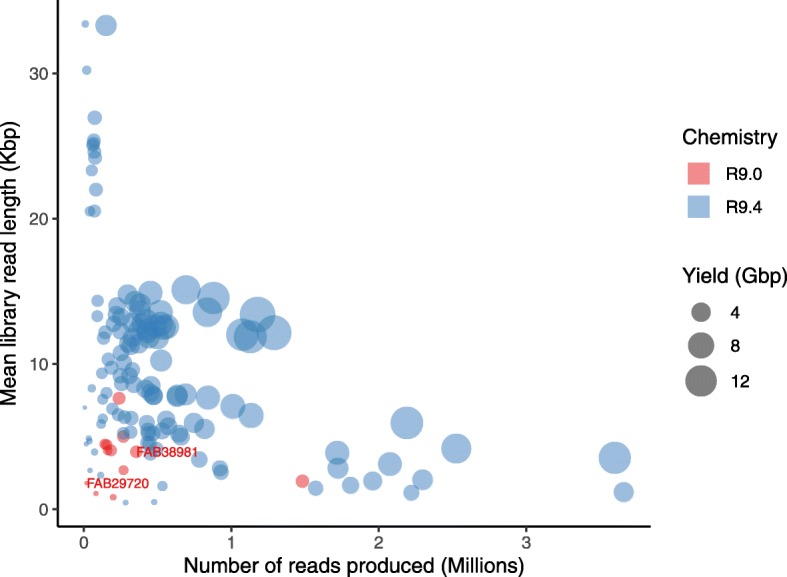
MinION throughput metadata. Relationship uniting library read length (average Kbp), number of read produced (in millions) and corresponding yield (in Gbp) per MinION flow cell. Data points obtained from the present study are labelled (justified right) with their respective flow cell number (FAB#). Lib_RAW_ was run of FAB29720 and Lib_GEL_ + Lib_MAG_ on FAB38981

Gathering published R9 and R9.4 MinION flow cell metadata from users showed that the number of reads produced per run and their average length follows a seemingly hyperbolic trend (1/x) (Fig. 6). As expected, R9.4 runs greatly exceeded the throughput of R9.0 runs from the increased speed of sequencing offered by this chemistry (250 to 450 bps, see introduction). In this plot, runs exhibiting some of the highest realized yield by users (up to > 15 Gb) are centrally located producing > 1 million reads with read average length > 12,000 bp. Nonetheless, high yield (> 12 Gbp) were also obtained for smaller fragment libraries of 2000–6000 bp average length producing > 2–3 million reads. Placing our runs in context with other R9 data point shows that they are dwarfed by current R9.4 output (Fig. 6).

## Discussion

### Nanopore sequencing

In early nanopore runs, we had assumed that our lack of experience in preparing sequencing libraries (and perhaps inappropriate manipulation of gDNA) was causing unwanted fragmentation, resulting in an excess of reads inferior to 1000 bp. However, thanks to guidance from ONT tech support, investigation of our original DNA extract’s integrity revealed an abundance of low molecular weight (LMW) fragments, which without mitigation were logically carried down through steps of library preparation and sequenced. Indeed, while ONT flow cells can sequence extremely long DNA fragments, the generated read length directly reflects the size distribution of fragments present in the input sequencing library, and thus LMW fragments need to be reduced as much as possible. With limited equipment available, we followed two simple mitigation strategies consisting of HMW fragments excision on gel electrophoresis (Lib_GEL_) or performing 0.4X magnetic bead wash (Lib_MAG_), both of which proved effective in diminishing LMW fragments (Fig. 3, Additional file 3: Figure S1). This was especially true for the gel excision methods, which however was also the most consuming DNA-wise (see Additional file 1). The 0.4X magnetic bead wash was also effective but less than the above.

Other than DNA integrity, increasing input library concentration seemed critical to improve run results but is difficult to sort out in the present study. Indeed, we not only worked without accurate concentration measurements (i.e. NanoDrop inaccuracy) but also with two flow cells of variable quality, the former of which (Lib_RAW_ run) had about two times less active nanopores at run start (611 vs. 1286) and also died faster (34 h vs. 44 h run). Nonetheless, it would appear than loading a higher volume of library improved the overall run throughput since our second flow cell (Lib_GEL_ + Lib_MAG_) produced > 200 reads/nanopore while the first one (Lib_RAW_) produced ~ 30 reads/nanopore (calculation based on total 1D reads presented in Table 1 and active nanopore numbers at run start). In this context, small research groups wishing to venture in nanopore sequencing should ensure that they can access a Qubit (or a similar platform) to maximize run throughput. Finally, the presence of inhibiting contaminants (e.g. proteins) is known to affect run performance [19]; however, our NanoDrop measurement suggested adequate DNA purity (absorbance at 260/280 and 260/230 of 2.11 and 1.97, respectively).

### Nanopore data and assemblies

Our testing of 2D ligation kits showed that a large proportion of template reads (1Df) did not have a matching complement (1Dr) reads, preventing the formation of consensus reads (2D) (see read numbers, Table 1). In some instances, the complement (1Dr) was sequenced, but 2D consensus still did not form, perhaps because of their lower quality or partial length (i.e. not fully overlapping with their 1Df). We do not know whether the above observations would apply to the more recent 1D^2^ libraries (R9.5 chemistry), whose sequencing strategy differs (hairpin-less libraries, see introduction), but users should probably expect lower output for such libraries (a trade-off for increased read accuracy). Considering the low number of 2D reads we obtained as compared to 1Df (> 32,000 vs. > 143,000 respectively, see Table 1), we decided to assess both read classes in assembly, especially following ONT’s commercial push toward 1D kits with chemistry R9.4. In preliminary analyses with Canu’s default parameters, assembly files solely included the CP genome of *C. ashmeadii* and very few bacterial contigs. This is because, by default, Canu only uses reads exhibiting >40X coverage for correction [20] and thus only the most abundant molecules get assembled. Here, we adjusted correction parameters to maximize read retention during correction (see methods) in order to ‘unlock’ Canu’s potential to assemble contigs with lower coverage. Doing so, Canu revealed a much greater diversity of contigs, including CP SVs as well as nuclear and bacterial contigs. The discovery of CP SVs also allowed us to gauge best error rate for corrections by monitoring assembly correctness with the above adjusted parameters (Additional file 14: Figure S12). For instance, we noted that for 2D data, loosening error rate led to the ‘stitching’ of SVs on the CP genome extremities (i.e. misassembly), preventing genome circularization. In contrast, 1Df assemblies seemed more robust to relaxed error rates, but this may be confounded by their much greater sample size rather than related to read quality.

### Organelle polishing and SNPs

Genome quality quickly increased through steps of the selected polishing pipeline. We favored first using native tools (Racon followed by Nanopolish) and then Illumina polishing (Pilon) to resolve any remaining errors. Our results focused on two organelles with different read coverage (and read class) also provided an opportunity to examine impact of such variable on final genome quality (Table 4, Additional file 11: Figure S9). Overall, we noted that while Racon offered some quality improvement, Nanopolish provided much of the ‘legwork’ (including greatly extending homopolymers, Additional file 2: Table S6) and Pilon fixed most (but not all) of the remaining errors. Both Nanopolish and Pilon seemed sensitive to read coverage since the quality of the polished 1Df MT genomes, which exhibited much lower read coverage than the CP genome (42X vs. 815X), remained lower with both tools (Table 4). However, when comparing the quality of the 1Df and 2D CP genome polished with high read coverage (815X vs. 215X), nearly identical results were obtained, suggesting that error correction may rapidly plateau with increasing coverage (note that for 2D reads, the actual signal coverage was ~430X rather than 215X since Nanopolish accesses both the signal of the 1Df and 1Dr underlying a consensus 2D read).

Within the polished genomes, we were also curious to investigate the quality of nucleotide stretches encoding for proteins (Table 4, Additional file 11: Figure S9) to investigate remaining substitutions or indels that may cause frameshifts and render annotation problematic. We determined that the quality of coding regions was generally higher than for the entire genome, and while native polishing resolved most substitutions, some indels remained (Table 4). The subsequent use of Illumina data was critical to further raise their quality to perfection (i.e. 100% identity, no indels or substitutions) in the high coverage CP genome but some indel/substitution errors remained in the lower coverage MT (> 99.8% identity, < 0.2% indels and substitutions, Table 4). Unfortunately, we could not pinpoint with certainty why coding regions exhibited slightly higher quality than the genomes, but the pattern does not appear related to homopolymer length (Additional file 2: Table S6) or overall homopolymer content (~ 16% in homopolymer > 4 bp in each of genome or genes, not shown).

Homopolymers represent a known issue of nanopore reads and assemblies. Here, we investigated Nanopolish default parameter, as well as the *--fix-homopolymer* option. Doing so, we observed that the last base of homopolymeric stretches was sometimes truncated and that the pattern was not consistent with one option or the other. Hence, we chose to combine polished results in a consensus by keeping all insertions (since deletions are more common in nanopore data, e.g. [21]). We also experimented feeding Nanopolish our final, manually curated CP genome and noticed that rather than converging to that answer, Nanopolish diminished genome quality. This demonstrated that with our data sets, we had reached the maximum polishing ‘potential’ that this tool can offer. Lastly, testing for SNPs presence, both Pilon and Nanopolish found numerous ambiguous calls; thus, combining results from these tools (i.e. to identify congruent SNPs position) may represent a safe practice. Unexpectedly, testing for SNPs with Nanopolish highlighted potential issues when polishing individual genomes from metagenomes because of the cross-mapping of conserved genes from different genomic compartments (e.g. CP *atp*A reads on MT *atp*1, Additional file 12: Figure S10) leading to spurious SNPs calls. Based on these observations, we advise that reads should probably be carefully sorted prior to polishing since conserved regions of different origin may exhibit sequence identity that lie within those of raw nanopore reads.

### Structural variation

Recovering SVs in short read next generation sequencing experiments is difficult because polymorphic regions may be flanked with conserved ones causing assembly fragmentation. In this context, long reads can greatly improve assemblies by ‘bridging’ the span of such complex genomic regions. An added advantage of long reads is that the assembled polymorphism, if within the length of generated reads, can also be verified at the raw read level. Unfortunately, while we could phase the entire *psb*A to ORF9 genomic region of the main chlorotype (Additional file 15: Figure S13), we could not determine the pattern of association of the SVs (Fig. 4b) on alternative chlorotypes with our modest data sets (sequencing depth insufficient). Although our discovery of SVs represents the first clear report of CP heteroplasmy in siphonous green algae, recent Illumina-based studies of multiple genera reported numerous partial genomes [22] indicating that the phenomenon may be widespread in this group of algae. Heteroplasmy was recently documented in the related Ulvophycean order Ulothrichales in *Capsosiphon fulvescens* (C.Agardh) Setchell & N.L.Gardner [23], but occurs from the flipping of the short single copy section (SSC) found between the large inverted repeat characteristic of numerous green lineages [24]. This polymorphim differs from the SVs observed here that exhibit presence/absence of intronic ORFs. Beyond the Ulvophyceae, heteroplasmy was also documented in the model unicellular green microalga *Chlamydomonas reinhardtii* P.A.Dangeard (Chlorophyceae) and the model brown macroalga *Ectocarpus siliculosus* (Dillwyn) Lyngbye (Phaeophyceae) [25, 26].

Potential mechanisms leading to heteroplasmy include biparental inheritance or de novo mutation [27]. Here, it is possible that the observed *psb*A intron polymorphism (Fig. 4b) is produced in vivo via homing endonucleases and/or retrohoming by reverse transcriptases as shown in *Chlamydomonas* spp. [28, 29], and as suggested by BLASTp reports (Additional file 16: Figure S14 and Additional file 17: Figure S15) of the corresponding ORFs in *C. ashmeadii*. Retrohoming corresponds to the reintegration of a transcribed intron into DNA by reverse splicing, while homing endonucleases repair double stranded breaks at specific recognition sites by copying the gene encoding themselves (i.e. the ORF and adjacent DNA) into the broken chromosome. Considering that *psb*A represents a critical polypeptide of the photosystem II that is translated at high rates in light conditions [30], we hypothesize that the above mechanisms could help maintain efficient repair/transcription/splicing of this gene or help modulate photosynthesis efficiency. We also hypothesize that recombination of interspersed repeats ASH1.1 and ASH1.2 into ASH1.3, which results in the excision of ORF8 (Fig. 4b), may be mediated by the presence of a palindromic site under putative control of an endonuclease that is specific to this recognition pattern (Additional file 13: Figure S11). However, BLASTp reports of these ORFs (ORF7, 8 and 9, Additional file 17: Figure S15) suggest their function as DNA primases/polymerases, thus in DNA/RNA synthesis rather than recombination. It is nonetheless possible that recombination occurs under the control of enzymes (ORFs) encoded elsewhere on the CP (i.e. upstream or downstream). Future resequencing may help reconstruct longer SVs and further decipher the mechanistic of DNA recombination in the CP genome. Long read transcriptomics may also shed light on transcription and splicing patterns.

Finally, using scanning electron and fluorescence microscopy, Miyamura and Nagumo [31] showed that both female and male gametes of *Caulerpa* carry one CP genome each, but that in male gametes it seemingly disappears before fusion. Our results cannot rule out paternal leakage but considering that 3 chlorotypes may be present in *C. ashmeadii* (Fig. 4b), some of this polymorphism may be produced in vivo (unless polymorphic CP chromosomes can be packaged within a single CP organelle, which is currently unknown). Our exploration of SNPs revealed a single unambiguous variable site and we expected that in the case of paternal leakage, many more SNPs would have been detected (note that the extent of CP SNPs polymorphism in *C. ashmeadii* population or any *Caulerpa* spp. is currently unknown). We did not detect SVs nor SNPs in the MT genome. Overall, single-cell genomic experiments [32] of isolated gametes may represent an interesting avenue of research to shed further light on all of the above.

### Bacterial diversity

We relied on the hybrid 1Df assembly (e.g. best N50, cumulative length and binning/COGs, Table 3, Additional file 8: Figure S6) as a reference hologenome to profile the abundance of the different genomic compartment/taxa (i.e. bins) via mapping. This analysis showed that while being of modest size, our nanopore 1Df and 2D reads tracked very closely profiles obtained with Illumina data (Fig. 5b); and thus, in future studies, when a reference holo/metagenome is available, long nanopore reads may also be used to examine community profile variation across multiple samples (e.g. across *Caulerpa*’s stolon vs. rhizoid or frond). Interestingly, and perhaps predictably, a larger proportion of 1Df (> 33%) than 2D reads (< 20%) were unclassifiable via mapping (Additional file 2: Table S7), probably because of their lower quality. Fine-tuning BWA-MEM parameters [33] may improve on this aspect, but in a metagenomic context, this may also increase the probability of lower quality reads mapping to unrelated regions. Among the six identified bacterial taxa, the Rodospirillaceae sp.1 recruited the most reads (Fig. 5b), which amounted those mapping to the CP and most bacterial taxa also recruited more reads than the MT (compare read counts in Table 2 vs. Additional file 2: Table S5). Nonetheless, because the CP and MT organelles represent small genomes as compared to bacterial or nuclear chromosomes, they are actually found in very high copy number in the siphonous cell (i.e. coverage, see Table 2).

The six taxa/familes identified are known as mutualistic symbionts able to photosynthesize (Rodospirillaceae, Rhodobacteraceae) (e.g. [34, 35]) or endosymbionts of siphonous green algae and the Streptophyta (Flavobacteriaceae and Phyllobacteriaceae) (e.g. [11, 36]) as well as pathogens (Cardiobacteriaceae) (e.g. [37, 38]). Here, considering the seemingly tight association of these families with host species and their abundance in the present hologenome, we hypothesize that most represent endosymbionts participating in metabolic function of *C. ashmeadii*’s siphonous cell, some of which may be obligate or facultative. Interestingly, previous accounts of associated bacteria with *Caulerpa* spp. using 16S metabarcoding have identified some of these families, but in much lower abundance (e.g. see [17, 39]). In these studies, authors made an effort to sterilize and DNA decontaminate the sample surface to reduce epiphytic DNA, and future whole genome sequencing of *Caulerpa* with long or short reads could follow such methodology. Completing bacterial genomes to circularized chromosomes with further nanopore reads may reveal taxa exhibiting genome size reduction and loss of function as generally observed for obligate (i.e. co-evolved) endosymbionts [40, 41]. Single cell genomics on isolated gametes, could also provide direct evidence of obligate bacterial endosymbionts transmitted vertically (e.g. see [42] for an account of endosymbionts observed within *Bryopsis hypnoides* gametes).

### Nuclear genome outlook

Using GenomeScope to estimate *C. ashmeadii*’s nuclear genome size (Additional file 7: Figure S5) from Illumina kmers led to an estimate of > 26 Mbp (including < 5 Mbp in repeat content). This estimate is congruent with the recent sequencing and hybrid assembly (PacBio + Illumina) of a *Caulerpa* species of economic importance in Japan (*C. lentillifera*, ~ 26 Mbp [43]). Interestingly, our best (cumulative) assembled length of > 24.4 Mbp (Table 3) is in very close range with the above estimates, indicating that the majority of the nuclear genome is present in our current assembly despite being fragmented. GenomeScope reported a repeat content estimate < 5 Mbp, while we measured >2Mbp in our best assembly (Table 3), indicating that an additional ~ 3 Mbp of repeats may remain to be resolved.

GenomeScope returned fairly low heterozygosity values for *C. ashmeadii*, suggesting that our sequenced specimen contains both maternal and paternal nuclear chromosomes. The life cycle of *Caulerpa* spp. is thought as gametic (also termed diplontic) but remains unclear because a recent study of indigenous and alien invasive species in the Mediterranean Sea found both free-living haploid and diploid thalli [44]. Considering that parthenogenetic development of female gametes that can germinate and grow into adult thalli is known in other gametic siphonous green alga such as *Codium* [45], the occurrence of haploid *Caulerpa* thalli in nature is still compatible with a gametic life cycle (rather than *Caulerpa* exhibiting a sporic life cycle with alternation of generations, also termed haplodiplontic). Polyploidy may also affect heterozygosity values and the above authors report that endopolyploidy (i.e. polyploidy of an organ within a diploid organism) may occur in the frond (the ‘pseudo-organ’ sequenced here) and that gametes were unreduced (i.e. polyploid) in *C. prolifera* (a close relative of *C. ashmeadii*). While endopolyploidy or autopolyploidy cannot be determined from the present genomic data, allopolyploidy is unlikely (i.e. hybridization was previously hypothesized in *Caulerpa* spp., [46]) since measured individual heterozygosity would have been much higher. Resequencing of the nuclear genome at higher coverage than presently available (Table 2) will allow the characterization of SNPs and eventual (chromosome-scale) structural variants to gain further insights into *C. ashmeadii* ploidy levels.

### Prospects and recommendations

Since the early days of the MinION Access Program in 2014, nanopore sequencing has seen rapid improvements, especially in the first two years of the program up to chemistry R9. While data output has increased drastically with R9.4 (Fig. 6), it would appear from the literature that data quality has only slightly increased since R9 (e.g. compare our Fig. 1 with Fig. 2 in [47]). ONT has announced the release of chemistry R10 (Summer 2019, www.nanoporetech.com), whose pores exhibit a longer barrel and dual reader head that will improve signal. With R10, ONT reports that raw read quality may be similar to R9.4 (L. Jayasinghe, plenary communication, ONT London Calling 2019, 05/24/2019) but that higher consensus accuracy of 99.999% can be reached because errors will be more random rather than systematic. Awaiting such improvements, our investigation shows that with current chemistries, indels may remain following signal polishing (Table 4, Additional file 11: Figure S9), which may still be problematic for automated annotation of genomes (i.e. gene prediction) when Illumina reads are not available for further correction. The issue is however not unique to nanopore assemblies since it was also recently reported from PacBio assemblies [48].

Our results also suggest that coverage of 40X may not be sufficient but that the quality of polished genomes may rapidly plateau with higher coverage. Perhaps sequencing depth of 100X maximizes signal polishing benefits/computing time (e.g. as used in [49]). In our opinion, using consensus 2D reads (now 1D^2^) in a (meta)genomic context seem to provide little advantage over 1D reads regarding assembly and final polished genome quality. 1D reads can also be produced in much greater abundance (Table 1) with simpler library preparation and bioinformatic logistics (i.e. ramification of files, folders and analyses). Finally, collected run metadata for 1D reads would indicate that to maximize read length/number on MinION flow cells (desirable for genomic projects), input libraries with fragment sizes > 10 kb may generate > 1 million reads (see central points, Fig. 6). Some of these libraries were prepared by shearing at ~ 20 kb following guidelines to optimize yield in nanopore runs [50, 51].

## Conclusions

In spite of early difficulties with library preparation due to LMW fragments, we successfully increased read size distribution with minimal equipment using two simple decontamination methods to generate read numbers and yields closer to those previously published for R9 flow cells (now dwarfed by R9.4 output, Fig. 6). We believe that our results, however, could have been further improved with access to a platform such as Qubit to measure accurate DNA concentration. Although flow cell quality was also an issue in our first R9 run (i.e. low active pore numbers), this problem has most likely been streamlined by ONT, especially since the commercial launch of the MinION platform. Here, the CP genome of *C. ashmeadii* could only be resolved as a circular molecule with nanopore assemblies, which also allowed the characterization of structural variants (SVs). Our modest nanopore data sets also resolved the MT genome as well as proved encouraging in a hybrid framework to improve assembly contiguity, binning and profiling of bacterial communities. We expect that deeper nanopore sequencing with current output will provide a wealth of bacterial and nuclear reads to bring these compartments closer to completion. Considering the portability, ‘run until’ capability (to test and control the output of different libraries), and opportunity to troubleshoot *in house* difficult samples that commercial facilities may not invest time in, the MinION provides unprecedented flexibility in DNA sequencing for small user groups working on emergent and non-model organisms, and with limited budgets.

## Methods

### Specimen

Total genomic DNA was extracted from live fronds (15 g blotted-dry) originating from a single individual of the *C. ashmeadii* holobiont collected in the Northeast Gulf of Mexico at Howard Park, Tarpon Springs, Florida (Pinellas county), USA (Specimen TS1851, date of collection: 09/22/2013, location: 28°09′14.5″N 82°48′24.1″W, depth: < 2 m) (Florida Fish and Wildlife Conservation Commission license #1000427446). DNA extraction followed the Dellaporta protocol [52]. Extraction was carried out in 8 volumes and precipitated DNA pellets were pooled prior to elution in order to yield large amounts of DNA (estimated > 1000 μg) as to not be limited for experiments and future resequencing. The herbarium voucher is maintained in the personal collections of the primary author.

### DNA clean-up and concentrations

Initial nanopore sequencing (library Lib_RAW_) resulted in numerous LMW fragments (i.e. reads < 1000 bp). To help rid LMW DNA fragments present in our DNA extract, we used two different approaches consisting of directly excising high molecular weight (HMW) DNA from an agarose gel (library Lib_GEL_), or performing a 0.4X magnetic bead purification (library Lib_MAG_) in order to preferentially bind HMW DNA (see Additional file 1 for further methodological details). Note that all DNA concentrations reported in the present study were measured via NanoDrop [53] and thus deviate from recommended concentrations measured by Qubit in ONT’s protocols. We were not aware of NanoDrop inaccuracy in early runs (i.e. DNA concentration overestimation) and thus in subsequent library preparation we purposely worked from larger readings and increased the amount of library loaded to the flow cell to improve output results. Following sequencing, we compared the read size distribution and flow cell output of the three libraries to assess effectiveness of the above LMW decontamination strategies.

### Library preparation

Our first library Lib_RAW_ was prepared according to the protocol for the 2D kit SQK-MAP006, with fragmentation of NanoDrop estimated ~ 1 μg DNA at 6 kb with a Covaris® g-TUBE™. Our second run with cleaned-up DNA (i.e. LMW-decontaminated), Lib_GEL_ (fragmented at 6 kb) and Lib_MAG_ (not fragmented), followed ONT’s protocol for the 2D sequencing kit SQK-NSK007 but starting with a larger amount of NanoDrop estimated DNA concentrations than above (increase to readings of ~ 2 μg and ~ 5 μg, respectively, which were probably still lower than recommended Qubit concentrations of 1 μg). Ensuing library preparation steps consisted in repairing DNA from putative damage (e.g. nicks) with the NEBNext® FFPE DNA Repair Mix (New England Biolabs, Ipswich, MA), followed by end-repair and dA-tailing with the NEBNext® Ultra™ II End Repair/dA-Tailing Module (New England Biolabs), and subsequent ligation of biotinylated ‘leader’ and ‘hairpin’ adapters with Blunt/TA Ligase Master Mix (New England Biolabs). Between enzymatic ‘repair’ steps, products were cleaned with AMPure® XP magnetic beads (in 1X ratio), while products of enzymatic ligation containing biotinylated adapters were cleaned with Dynabeads® MyOne™ Streptavidin C1 (ThermoFisher Scientific Inc.). DNA libraries bound to C1 beads were eluted in 25 μL of ONT’s buffer. The sequencing-ready eluted libraries had NanoDrop readings of > 220 ng/μL for Lib_RAW_, > 400 ng/uL for Lib_GEL_ and > 280 ng/uL for Lib_MAG_, which as mentioned previously may deviate from ONT’s recommended Qubit DNA concentration of > 200 ng/μL.

### Nanopore sequencing

Lib_RAW_ was loaded on the first flow cell (ID# FAB29720) with 6 μL every 12 h (according to ONT protocols at the time) and run with default script FLO-MAP103 on the Mk I MinION™ until no further reads were produced (> 34 h). Because Lib_RAW_ resulted in very low output (low read counts and short reads caused by both LMW and low input DNA concentration, i.e. NanoDrop), libraries Lib_GEL_ and Lib_MAG_ were each loaded on the second flow cell (ID# FAB38981) with their entire eluted volume of 25 μL, while adjusting the amount of H_2_O in the final loading mix. Lib_GEL_ was loaded first and the run started with the default run script FLO-MIN105 on the MkI B MinION™. Following 6 h of sequencing, the run was stopped, the flow cell refreshed with ONT’s wash kit and Lib_MAG_ was loaded. The run was then restarted with the same default script until no further reads were produced (> 44 h). Platform QC prior to loading Lib_RAW_ on the first flow cell indicated a total of 611 nanopores (307, 191, 89, and 24 nanopores allocated to well-groups). Platform QC prior to loading Lib_GEL_ on the second flow cell indicated a total of 1286 viable nanopores (with 501, 435, 266 and 84 ‘mux-scan’ allocated nanopores in well-groups 1 to 4), which prior to loading Lib_MAG_ had decreased to 503 nanopores (372, 188, 52 and 10 nanopores allocated to well-groups). All libraries were basecalled with Metrichor (agent 2.3.8.3) in the cloud (metrichor.com) with the corresponding - script (see above). Downloaded *fast*5 files containing signal-level information and basecalled reads were then processed with Poretools [54] to extract consensus 2D and 1D reads in *fastQ* format (template and complement reads are referred as 1Df and 1Dr throughout, i.e. for forward and reverse). Although Metrichor classifies reads as ‘pass’ and ‘fail’ (corresponding to higher and lower quality reads according to a mean base quality score Q > 9), we used all data available to maximize read numbers.

### Nanopore assemblies

Prior to assembly, we concatenated each of the 1Df and 2D *fastQ* data sets (Lib_RAW_ + Lib_GEL_ + Lib_MAG_) and filtered them for reads > 1000 bp, the default minimum read size for data input in the Canu v1.4 assembler [20] (we chose to exclude complement 1Dr reads in order to test the prospects of template 1Df reads for de novo assembly as would be generated by more recent 1D kits SQK-LSK109; likewise, the behavior of consensus 2D reads in assembly may be similar to newer 1D^2^ library preparation SQK-LSK309). Read correction was conducted with Canu’s built-in algorithm set with non-lossy (low coverage) parameters in order to retain a maximum number and diversity of reads from the *Caulerpa* hologenome (e.g. *corMhapSensitivity =* high, *corMinCoverage =* 0 and *corOutCoverage =* 4000; the latter calculated as to exceed the total number of bases/number of reads of the corresponding dataset). A single round of corrections was conducted for 2D reads while 1Df reads were subjected to three rounds and trimmed according to Canu’s default parameter. The subsequent assembly step of the corrected/trimmed reads into contigs (Canu produces contigs rather than scaffolds) was performed with the error rate parameter set to 2.5% for 1Df reads and 1.5% for 2D reads, as recommended for R9 data [20] (*errorRate* of 0.015 and 0.025 in Canu v1.4; note that more recent versions of Canu (i.e. v1.8) use a new parameter *correctedErrorRate* and values to input may differ, see https://github.com/marbl/canu).

### Hybrid and short-read assemblies

A total of 27.5 million Illumina read pairs (2 × 150 bp) were generated commercially by MRDNA (www.mrdnalab.com, Shallowater, TX, USA) on a HiSeq 2500 platform (Illumina Inc., San Diego, CA, USA). Short read-only scaffolds were assembled in SPAdes v3.10.1 [55] with recommended options for 2 × 150 bp dataset including the BayesHammer error correction module, increasing kmer length (*−k* of 21, 33, 55, 77, 99 and 127), the *--careful* option, and the post-processing tool MismatchCorrector (used to minimize the number of mismatches in the final contigs). No preliminary trimming or filtering of was performed as recommended by the Spades team. For hybrid assemblies, we added the --*nanopore* flag to feed R9 1Df reads or 2D *fastQ* reads > 1000 bp.

### Binning

We conducted metagenome binning with MyCC [18] (parameters ‘*4mer*’ and ‘-*lt* 0.8’ and for contigs > 1000 bp) on the Illumina and hybrid assembly files to sort out genomic compartments and members of the *C. ashmeadii* holobiont consortium. As an indication of assembly and binning quality, we examined the total number of COGs reported by MyCC and performed reciprocal BLASTn across assemblies (BLAST suite v2.5.0+). Bacterial species (BACT) and nuclear (NU) scaffolds were identified with BLASTp [56] using COGs reported by MyCC (Additional file 10: Figure S8). We also explored the use of MyCC on nanopore assemblies, although this program is not optimized for such data.

### Hologenome profiles

To quantify the prevalence of *C. ashmeadii*’s genomic compartments in the different data sets (Illumina and ONT 1Df and 2D), we performed mapping on scaffolds binned from the best assembly (PE+1Df). To do so, raw reads were aligned with BWA-MEM v0.7.15 (bio-bwa.sourceforge.net/) with default parameters for Illumina reads or the *-x* flag for nanopore reads, and further sorted with Samtools v1.3.1 (samtools.sourceforge.net/) to obtain counts from uniquely mapped reads per contig/scaffolds for each bin/taxa. For the paired-end Illumina dataset, computations were done from a *.bam* file containing proper read pairs and no secondary alignments (samtools flags *‘-f 2 -F 2308*’). For each of the single-end nanopore 1Df and 2D data sets, computations were done for mapped reads excluding secondary alignments (samtools flag ‘*-F 2308’*). Finally, we used the Samtools *depth* function to determine realized read coverages.

### Genome size and repeats

To assess *C. ashmeadii*’s nucleus size, heterozygosity and repeat length, we used GenomeScope (qb.cshl.edu/genomescope/ [57]); however, because this program is designed for eukaryotic organism, we sorted the Illumina dataset to deplete bacterial reads (i.e. exclusion of reads mapping to bacterial scaffolds) (Additional file 7: Figure S5). We then counted kmers of length 19 with Jellyfish v2.2.6 [58] to establish a histogram file for input to GenomeScope, which was run with a read length parameter of 150 and max kmer coverage of 10,000 (to estimate the maximum genome size by using the entire kmer distribution). Finally, to gain further perspectives on assembled repeat content (length and type) and benefits of using nanopore data to improve repeat assembly, we also run the de novo repeat family discovery tool RepeatModeler v1.0.11 (www.repeatmasker.org/RepeatModeler/) on scaffolds from the Illumina and hybrid assemblies (bacterial or nuclear scaffolds of min. Size 1000 bp). We also run Tandem Repeat Finder (TRF) v4.09 [59] to quantify larger tandem repeats than those reported by RepeatModeler (restricted to 1–5 bp motifs).

### Assembly polishing

We used native and hybrid polishing tools on the CP and MT genomes retrieved from the assembly files. After their circularization (i.e. by finding the overlap between the edges of their respective contigs via BLASTn), polishing was performed with Racon v0.5.0 (a native nucleotide-level consensus tool [60]) followed by Nanopolish v0.5 (a native signal-level polishing tool [4]) and finally Pilon v1.20 (a hybrid nucleotide-level polishing tool based on Illumina short reads [61]). Racon was run with three polishing rounds on the Canu contigs. Nanopolish was run with and without the --*fix-homopolymers* option on the racon contigs, and a consensus created from the two resulting contigs (i.e. keeping all observed base pair insertions). Pilon was then run with merged Illumina paired-ends using the single-end option (*−-unpaired* rather than *--frag*) on the nanopolished contig. To establish a final curated organellar genomes, we further compared the Pilon contigs with matching segments of Illumina and hybrid scaffolds to correct any remaining point insertions/deletions. Quality improvement of the genomes and protein-encoding genes was computed through polishing steps against the final curated version via BLASTn (as in the *Data quality* section further below). We also investigated for the presence of single nucleotide polymorphisms (SNPs) with Nanopolish (−-*snps*) and Pilon (−-*variant*). No polishing was attempted on BACT and NU genomes, whose nanopore coverage was extremely low and uneven (<5X and < 1X, respectively).

### Organelle annotation

Annotation of the genomes and CP structural variations were carried out in Geneious v11.1.5 (www.geneious.com [62]). Annotations were transferred from published organellar genomes or created by predicting ORFs. The translation frame and gene boundaries of protein-coding genes were carefully inspected and verified via BLASTp and BLASTx, while ribosomal genes were investigated via BLASTn aided by predictions from the RNAmmer 1.2 Server [63]. tRNAs were annotated with tRNAscan-SE v2.0 [64] and ARAGORN v1.2.38 [65]. The presence of interspersed repeats was investigated by aligning genomes on themselves via BLASTn. We predicted regions harboring short repeats with TRF v4.09 [59] and inverted repeats were checked with einverted and keeping motifs > 60 bp [66]. We used genoplotR v0.8.9 [67] to graph a summary linear view of the complete CP and MT genomes and the discovered CP SVs. A more detailed visual of the gene content of these circular genomes was graphed in OGDRAW v1.3.1 [68] and is available in the Additional file 5: Figure S3 and Additional file 6: Figure S4.

### Data quality

We used ‘loose’ BLASTn parameters maximizing alignment length of error prone data (*−word size* 7 *-reward* 1 *-penalty* − 1 *-gapopen* 2 *-gapextend* 2 *-dust* no *-soft_masking* false) to determine nucleotide identities and underlying indels (insertions/deletions) and substitutions (all brought to percentage values). To avoid ambiguous read alignments that may affect these estimates, we used a portion of the CP genome devoid of structural variations or complex regions (a segment of 70,840 bp in full agreement with the Illumina-only contig). The same parameters were used for the presented retrospective analysis (Fig. 1) of raw read quality improvement based on our data collected through the MinION Access Program (2014–2016) from chemistry R6 (SQK-MAP001) to R7/7.3 (SQK-MAP002/SQK-MAP003/SQK-MAP006) and R9, and per read type (1Df, 1Dr and consensus 2D reads). Since our R6 CP reads were insufficient in numbers for graphing, we matched R6 reads against the Lambda phage spike-in (48,502 bp) and control DNA CS (3560 bp segment of *E. coli* genome) that were used in our first runs (provided by ONT in the MAP).

### Output

Finally, to place our modest (suboptimal) R9 throughput results into context with those realized by other MinION users with the same or newer chemistries (R9.4), we combed through open access nanopore publications to extract run metadata. Namely, we specifically looked for studies that reported 1D run statistics per flow cells (for any sequencing applications, e.g. amplicon metagenomics, transcriptomics or shotgun genomics) and gathered the number of reads and base pair output (i.e. the sum of the length of all reads) as well as the average read length generated. If the study used 2D consensus library preparation, we gathered statistics for 1Df +1Dr reads to reflect total achieved 1D read throughput (as if a R9.4 1D library preparation kit had been used). Some run metadata were also obtained from the MinION forum ‘Poreboard’ competition where users report maximum achieved throughput on MinION flow cells. The gathered metadata is available online (github.com/tomsauv) for users wishing to plot their run performance.

### Computing

The MinION runs with chemistry R6 to R9 (2014 to 2016) were performed at Univ. of Louisiana at Lafayette on a Windows 7 PC (64-bit) with an Intel® Core™ i7-4770K (3.5 GHz) with 16 GB of RAM, 250 Gb SSD (with an overflow 3 Tb HDD for data storage). Data analyses, assemblies and polishing tools were run on a 40 cores Red Hat Linux server with a limit of 200 GB of RAM at the Smithsonian Marine Station (SMS). Plots were produced in R with package ggplot2 [69].

## Supplementary information

**Additional file 1.** Supplementary methods.

**Additional file 2.** Supplementary tables.

**Additional file 3: Figure S1.** Read length distribution. Density plot depicting read length improvement following decontamination of low molecular weight fragment (LMW). Lib_RAW_: original DNA extract, Lib_GEL_: Gel excised HMW DNA, and Lib_MAG_: HMW DNA selected via 0.4X magnetic bead wash. Data is shown for 1Df and 2D reads. Note the broader shoulder of Lib_GEL_ for sequence > 2500 bp.

**Additional file 4: Figure S2.** Assembly file content. Contig/scaffold abundance per genomic compartment across nanopore and hybrid assemblies. Bubble size represent the percentage of uniquely mapped reads to a given assembly and corresponding dataset (those representing < 1% no shown for figure clarity). Note that due to layering of the plot, numerous scaffolds < 20,000 bp in the Illumina and hybrid assemblies become hidden, thus two plots are shown to emphasize (a) bacterial scaffolds or (b) nuclear scaffolds.

**Additional file 5: Figure S3.** Circular Chloroplast genome map.

**Additional file 6: Figure S4.** Circular Mitochondrion genome map.

**Additional file 7: Figure S5.** GenomeScope profile. Kmer coverage and log coverage for the entire Illumina dataset (a and b), the dataset depleted of bacterial reads (c and d), and the dataset depleted of nuclear reads (e and f). Note the drop in kmer frequencies indicated by arrows and circles to point at the depletion of bacterial kmers overlapping with nuclear ones (a and c) and their recovery in the dataset depleted of nuclear reads (e). Note as well the successful depletion of the main bacterial kmer peak (*) in the bacterial depleted dataset (a and e vs. c).

**Additional file 8: Figure S6.** Bin reciprocity. Sankey plot depicting MyCC bin correspondence across Illumina and hybrid assembly files (Illumina PE + 1Df or 2D) and their taxonomy/origin in the *Caulerpa ashmeadii* holobiont based on COGs. Flow size linking bins represent the number of common scaffolds in the assembly files compared via reciprocal BLASTn. Numbers adjacent to bins represent the number of COGs reported by MyCC. Note the consistent delimitation of nuclear contigs but some instability in the binning of the Phyllobacteriaceae sp. and Rhodospirillaceae sp2. scaffolds. The Sankey diagram was built on the basis of html code available from Google Charts at https://developers.google.com/chart/interactive/docs/gallery/sankey. PE = Illumina Paired-Ends scaffolds, PE+1Df = hybrid 1Df scaffolds, PE+2D = hybrid 2D scaffolds.

**Additional file 9: Figure S7.** Metagenome binning. Comparison of delimited bins with MyCC (4mer) for three assemblies. (a) Experimental binning of Canu 1Df contigs. (b) Binning of Spades’ hybrid Illumina+1Df assembly. (c) Binning of Spades’ Illumina-only assembly. Bins containing nuclear and repeat contigs are emphasized with dashed circles. Remaining bins correspond to bacterial taxa. Note that printed bin numbers are unrelated between plots.

**Additional file 10: Figure S8.** Phylogeny of valyl-tRNA ligase. Exploratory phylogeny of valyl-tRNA ligase (COG0525) amino acid sequences extracted from nuclear and bacterial bins by MyCC. For taxonomic identification of bins, sequence context was retrieved via BLASTp against GenBank’s non-redundant protein databases. Note the occurrence of two different valyl-tRNA ligases in *Caulerpa ashmeadii* nuclear genome.

**Additional file 11: Figure S9.** Polished quality of protein-encoding gene. Detailed distributions of gene quality following polishing steps as (a) boxplot or (b) violin plot. For comparison, numbers reported between parenthesis in Table 4 correspond to the cumulative percentage identity, indels and substitutions for all genes, while the data points and distribution in the present figure represent individual gene values. Note the perfect quality of chloroplast genes following Pilon polishing. CP_1DF=Chloroplast genes from 1Df assembly, CP_2D = Chloroplast genes from 2D assembly, MT_1Df = Mitochondrion genes from 1Df assembly.

**Additional file 12: Figure S10.** BLASTn report for *atp*1/*atp*A. BLASTn alignment of mitochondrial *atp*1 and chloroplast *atp*A. Note identity level nearing ~ 70%, causing mismapping of low quality *atp*A reads on *atp*1.

**Additional file 13: Figure S11.** Putative chloroplast recombination mechanism. (a) Edited BLASTn report showing identity and common directionality of the interspersed repeats ASH1.1 and ASH1.2 and palindrome GTTTAAAC (italicized and boxed) acting as a potential endonuclease restriction site. Color coding represents boundaries of the putative excised (blue) and recombined genomic segments (green and red). Note that the blue fragment belonging to ASH1.2 on top extends up to the red fragment on the bottom on a physical distance of > 4600 bp up to ASH1.1 and ORF9 and exhibit the same directionality (i.e. see coordinates value) (b) Putative recombined repeat ASH1.3 at the palindromic site (green and red). Note that following recombination, the start codon and thus translation frame of ORF9 (Met for Methionine) is unaffected.

**Additional file 14: Figure S12.** Error rate vs. assembly. Impact of Canu’s error rate parameter on assembly of the circular chloroplast genome in the presence of structural variation (SV) (black bubbles). Note the occurrence of misassemblies (i.e. polymorphism/SVs stitched on the contig’s extremities) when using relaxed error rates with 2D data, while 1D assembly still produces a circularizable contig (i.e. no misassembly).

**Additional file 15: Figure S13.** Chloroplast genome long reads’ pile-up.

**Additional file 16: Figure S14.** BLASTp report for *psb*A intronic ORFs.

**Additional file 17: Figure S15.** BLASTp report for ORF7, ORF8, and ORF9.

## Data Availability

Raw nanopore and Illumina data are available under NCBI’s BioProject PRJNA515488. The annotated mitochondrion (MT) and chloroplast (CP) genomes and discovered chloroplast structural variants (SVs) were deposited in Genbank under accessions MH745227-MH745231.

## Abbreviations

BACT: Bacterial
COG: Clusters of orthologous groups
CP: Chloroplast
HMW: High molecular weight
Kbp: Kilo base pair
LMW: Low molecular weight
MAP: MinION Access Program
Mbp: Mega base pair
MT: Mitochondrion
NU: Nuclear
ONT: Oxford Nanopore Technology
ORF: Open reading frame
QC: Quality control
SMS: Smithsonian marine station
SNP: Single nucleotide polymorphism
SV: Structural variant/variation
TRF: Tandem repeat finder
