# Supplementary material for: Promising prospects of nanopore sequencing for algal hologenomics and structural variation discovery
Source: BMC Genomics. 2019 Nov 13;20:850. doi: 10.1186/s12864-019-6248-2 (PMC6854639; doi:10.1186/s12864-019-6248-2)

Additional file 1: Supplementary methods

Note that DNA concentrations reported below (and in the main manuscript) correspond to NanoDrop readings and thus deviate from recommended concentrations listed in ONT protocols, which are measured by Qubit (or equivalent). We were not aware of NanoDrop inaccuracy prior to preparing Lib_{RAW} and thus we purposedly worked from larger readings for the preparation of Lib_{GEL} and Lib_{MAG}, and increased the amount of library loaded to the flow cell for sequencing (see methods and data description).

Raw library (Lib_{RAW})

Raw DNA was fragmented to a length of ~6 kb in a total of 150 µL (20 µL DNA +130 µL H_2O) in a single Covaris® g-TUBE™ (Covaris, Inc. Woburn, MA) via centrifugation at 13,200 rpm for 30 seconds. DNA recovery was ~1 µg for use in library preparation for Nanopore sequencing.

Gel excision library (Lib_{GEL})

Raw DNA was fragmented to a length of ~6 kb in a total of 150 µL (20 µL DNA +130 µL H_2O) in a single Covaris® g-TUBE™ (Covaris, Inc. Woburn, MA) via centrifugation at 13,200 rpm for 30 seconds. The fragmented DNA was then loaded in eight wells of a 1% TAE gel containing 0.2 µg/mL EtBr (18 µL of fragmented DNA per well + 4 µL of 6X DNA loading dye, ThermoFisher Scientific Inc., Waltham, MA). The HMW DNA >4 kb (as indicated by a DNA ladder) was then excised manually from the gel on a UV transillumінator (Labnet International, Inc., Edison, NJ), with the agarose bands pooled and cleaned on a single column with the GenCatch™ Gel Extraction Kit (Epoch Life Science, Inc, Sugar Land, TX) in a final elution volume of 50 µL H_2O. DNA recovery was ~2 µg for use in library preparation for Nanopore sequencing, corresponding to an estimated ~30X loss from the original DNA amount.

Magnetic beads library (Lib_{MAG})

Raw DNA (no fragmentation performed) was mixed with AMPure® XP beads (Beckman Coulter, Indianapolia, IN) in a 0.4X ratio (20 µL DNA + 8 µL beads) and incubated for 5 min. The beads were then pelleted with a magnet and the supernatant containing the LMW fragments discarded. The HMW fragments bound to the beads were washed twice with 70% EtOH, air dried for ~30 seconds, and eluted in 50 µL H_2O. DNA recovery was ~5 µg for subsequent library preparation for Nanopore sequencing, corresponding to a >10X loss from the original DNA amount.

Gel electrophoresis QC

Lane L: DNA Ladder
Lane 1: gDNA with LMW contaminant (Lib_{RAW})
Lane 2: Gel-excised HMW gDNA (Lib_{GEL})
Lane 3: 0.4X supernatant LMW gDNA
Lane 4: 0.4X bead bound HMW gDNA (Lib_{MAG})

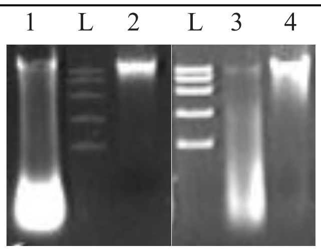

Supplement: Supplementary file 1 — Additional file 1. Supplementary methods. [file 12864_2019_6248_MOESM1_ESM.pdf]
