# Supplementary material for: Promising prospects of nanopore sequencing for algal hologenomics and structural variation discovery
Source: BMC Genomics. 2019 Nov 13;20:850. doi: 10.1186/s12864-019-6248-2 (PMC6854639; doi:10.1186/s12864-019-6248-2)
Supplement: Supplementary file 2 — Additional file 2. Supplementary tables. [file 12864_2019_6248_MOESM2_ESM.pdf]

Additional File 2: Supplementary tables

Table S1. Read length summary statistics. LibGEL values are bolded as best among the three libraries.

		Min.	1st Qu.	Median	Mean	3rd Qu.	Max.[1]
1Df	Lib_{RAW}	5	273	799	1,945	2,231	68,690
	**Lib_{GEL}**	5	**1,216**	**2,787**	**3,287**	**4,781**	41,490
	Lib_{MAG}	5	944	1,833	2,685	3,483	110,700
1Dr	Lib_{RAW}	5	79	298	1,539	1,146	127,800
	**Lib_{GEL}**	5	**581**	**1,897**	**2,428**	**3,808**	86,160
	Lib_{MAG}	5	271	929	1,656	2,024	156,600
2D	Lib_{RAW}	112	476.2	859	1,777	2,370	15,890
	**Lib_{GEL}**	103	**2,343**	**3,882**	**3,942**	**5,324**	50,120
	Lib_{MAG}	96	1,200	1,987	2,688	3,444	22,020

[1] Mappability of reads exhibiting maximum size not verified.

Table S2. Summary of scaffold repeat content. Estimated repeat length in total base pairs masked and as percentage of total scaffold length for a given assembly, as determined by RepeatModeler (RepeatM) and Tandem repeat Finder (TRF). The highest values indicative of improved repeat assembly/detection are in bold. Field representing total for a given repeat category or grand total are shaded in gray. PE=Paired-ends, LTR=Long Terminal Repeats.

		**Nuclear**			**Bacterial**		
**Software**	**Type**	**PE**	**PE+1Df**	**PE+2D**	**PE**	**PE+1Df**	**PE+2D**
**RepeatM**	**LTR elements**	49,857 bp<br>0.20%	**114,948 bp**<br>**0.47%**	26,768 bp<br>0.11%	16,954 bp<br>0.08%	**57,825 bp**<br>**0.28%**	22,089 bp<br>0.11%
	**DNA elements**	--<br>--	--<br>--	--<br>--	95,826 bp<br>0.47%	**161,932 bp**<br>**0.79%**	109,268 bp<br>0.53%
	**Unclassified**	1,316,012 bp<br>5.41%	**1,387,132 bp**<br>**5.67%**	1,310,929 bp<br>5.39%	**794,772 bp**<br>**3.89%**	771,693 bp<br>3.74%	738,427 bp<br>3.59%
	**Total Interspersed**	1,365,869 bp<br>5.61%	**1,502,080 bp**<br>**6.14%**	1,337,697 bp<br>5.50%	907,552 bp<br>4.44%	**991,450 bp**<br>**4.81%**	869,784 bp<br>4.23%
**RepeatM**	**Simple Repeats**	77,764 bp<br>0.32%	**94,222 bp**<br>**0.38%**	91,953 bp<br>0.38%	69,777 bp<br>0.34%	**70,476 bp**<br>**0.34%**	63,546 bp<br>0.31%
	**Low complexity**	**32,620 bp**<br>**0.13%**	31,321 bp<br>0.13%	29,453 bp<br>0.12%	10,083 bp<br>0.05%	10208 bp<br>0.05%	**11,437 bp**<br>**0.06%**
**TRF**	**Tandem**	462,429 bp<br>1.90%	**466,121 bp**<br>**1.90%**	444,991 bp<br>1.83%	295,152 bp<br>1.44%	304,614 bp<br>1.48%	**306,457 bp**<br>**1.49%**
	**Total Simple +Low complexity**	572,813 bp<br>2.35%	**591,664 bp**<br>**2.41%**	566,397 bp<br>2.32%	375,012 bp<br>1.83%	**385,298 bp**<br>**1.87%**	381,440 bp<br>1.85%
	**Grand Total**	1,938,682 bp<br>7.96%	**2,093,744 bp**<br>**8.55%**	1,904,094 bp<br>7.82%	1,282,564 bp<br>6.27%	**1,376,748 bp**<br>**6.68%**	1,251,224 bp<br>6.08%

Table S3. Genomescope report. Nuclear genome properties determined by Genomescope v1.0 using the Illumina dataset depleted from bacterial reads (see Fig. S5 panel c and d).

Property	min	max
**Heterozygosity**	0.409485%	0.416116%
**Genome Haploid Length**	26,268,957 bp	26,280,525 bp
**Genome Repeat Length**	4,867,943 bp	4,870,086 bp
**Genome Unique Length**	21,401,014 bp	21,410,439 bp
**Model Fit**	97.5292%	99.2466%
**Read Error Rate**	1.49446%	1.49446%

Table S4. Bin statistics. Summary statistics for MyCC delimited bins including number of scaffolds (N), N50 value and cumulative assembled length per bin. The most abundant bacterial species is shown in bold (see Table S4 as well).

Bin	N	N50 (bp)	Length (bp)
**Phyllobact. sp**	361	40,171	5,543,965
**Cardiobact. sp**	380	35,084	3,321,801
**Rhodospir. sp1**	**149**	**64,778**	**3,083,293**
**Flavobact. sp**	260	51,125	3,064,411
**Rhodobact. sp**	115	50,066	2,727,236
**Rhodospir. sp2**	37	187,044	2,656,942

Table S5. Bacterial read counts, relative abundance and coverage per dataset. Read counts and mean coverage were determined via mapping on the most contiguous assembly (PE+1Df). The relative abundance of bacterial species was computed from total bacterial read counts in a given dataset. Mapping of nanopore data was conducted with filtered reads (>1000 bp). Numbers in parenthesis represent standard deviation. Note low and uneven coverage for all species with current data. Nonetheless, the Rhodospirillaceae sp1 is the only species whose mean coverage exceeds its standard deviation confirming its important abundance in the *C. ashmeadii* holobiont. Value reported underlay those printed for the 'Bacteria' field in Table 2.

	**1Df**	**2D**	**Illumina PE**
**Cardiobacteriaceae**	4,142	1,245	1,058,744
	8.35%	9.03%	9.54%
	5.4X (± 22.1X)	4.1X (±13.8X)	136.0X (±571.0X)
**Flavobacteriaceae**	4,123	1,449	1,222,835
	8.31%	10.51%	11.02%
	3.7X (±4.6X)	2.3X (±2.7X)	124.1X (±196.3X)
**Phyllobacteriaceae**	5,342	1,379	1,181,104
	10.76%	10.00%	10.65%
	3.3X (±14.0X)	2.0X (±7.9X)	85.1X (±310.6X)
**Rhodobacteraceae**	2,893	771	758,879
	5.83%	5.59%	6.84%
	3.2X (±5.7X)	1.8X (±3.9X)	108.4X (±305.6X)
**Rhodospirillaceae sp.1**	**30,142**	**8,144**	**6,067,051**
	**60.73%**	**59.07%**	**54.68%**
	**24.8X (±12.3X)**	**8.8X (±5.2X)**	**599.2X (±348.6X)**
**Rhodospirillaceae sp.2**	2,988	798	806,256
	6.02%	5.79%	7.27%
	3.2X (±13.5X)	1.8X (±8.4X)	103.3X (±226.6X)

Table S6. Distribution of homopolymer length per length class. Among homopolymer proportion (%) through polishing steps. Note the important extension (i.e. correction) of homopolymers superior to 5 bp following signal polishing with Nanopolish (in bold). Values are reported for all stretches >4 bp found in the 1Df nanopore-assembled chloroplast genome.

**CP_1Df**		**4 bp**	**5 bp**	**6 bp**	**7 bp**	**8 bp**	**9 bp**	**≥10 bp**
**Genome**	**Canu**	79.42	20.53	--	--	0.05	--	--
	**Racon**	65.81	33.38	0.34	0.18	0.24	--	0.05
	**Nanop.**	47.55	26.30	**13.55**	**6.20**	**3.82**	**1.39**	**1.20**
	**Pilon**	46.57	24.82	**13.01**	**7.21**	**4.22**	**2.36**	**1.82**
**Gene**	**Canu**	79.47	20.53	--	--	--	--	--
	**Racon**	66.28	33.27	0.25	0.06	0.13	--	--
	**Nanop.**	47.52	26.41	**13.89**	**6.78**	**3.73**	**0.93**	**0.74**
	**Pilon**	46.44	24.89	**13.42**	**7.91**	**4.14**	**2.10**	**1.11**

Table S7. Mapped vs. unmapped read counts and proportion per dataset. Number and relative abundance of reads mapped to the most contiguous assembly (PE+1Df) regardless of genomic compartment.

	**1Df**	**2D**	**PE**
**Mapped**	95,563	25,844	49,435,868
	66.46%	80.32%	93.35%
**Unmapped**	48,234	6,333	3,519,650
	33.54%	19.68%	6.65%
**Total**	143,797	32,177	52,955,518
	100%	100%	100%
