# Supplementary figures and images for: Promising prospects of nanopore sequencing for algal hologenomics and structural variation discovery
Source: BMC Genomics. 2019 Nov 13;20:850. doi: 10.1186/s12864-019-6248-2 (PMC6854639; doi:10.1186/s12864-019-6248-2)

Density

1Df

2D

LibRAW  
LibGEL  
LibMAG

0 2500 5000 7500 10000 0 2500 5000 7500 10000

Read length (bp)

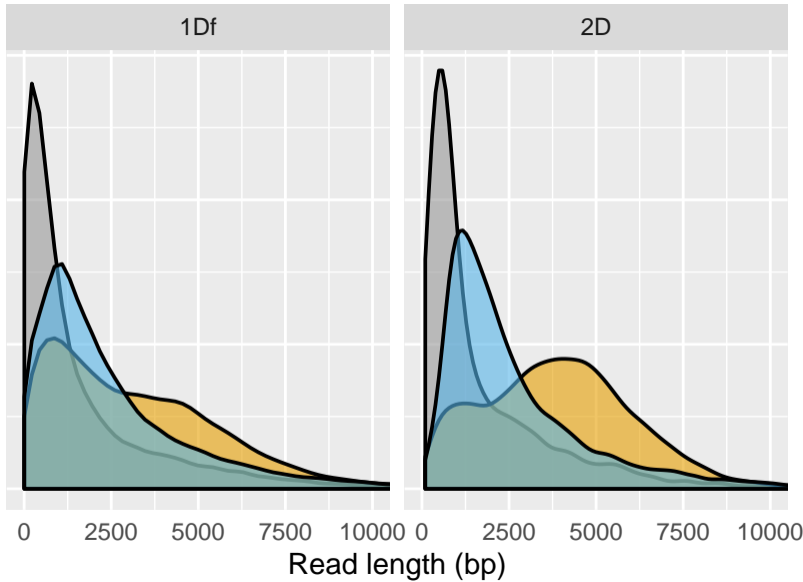

Supplement: Supplementary file 3 — Additional file 3: Figure S1. Read length distribution. Density plot depicting read length improvement following decontamination of low molecular weight fragment (LMW). LibRAW: original DNA extract, LibGEL: Gel excised HMW DNA, and LibMAG: HMW DNA selected via 0.4X magnetic bead wash. Data is shown for 1Df and 2D reads. Note the broader shoulder of LibGEL for sequence > 2500 bp. [file 12864_2019_6248_MOESM3_ESM.pdf]

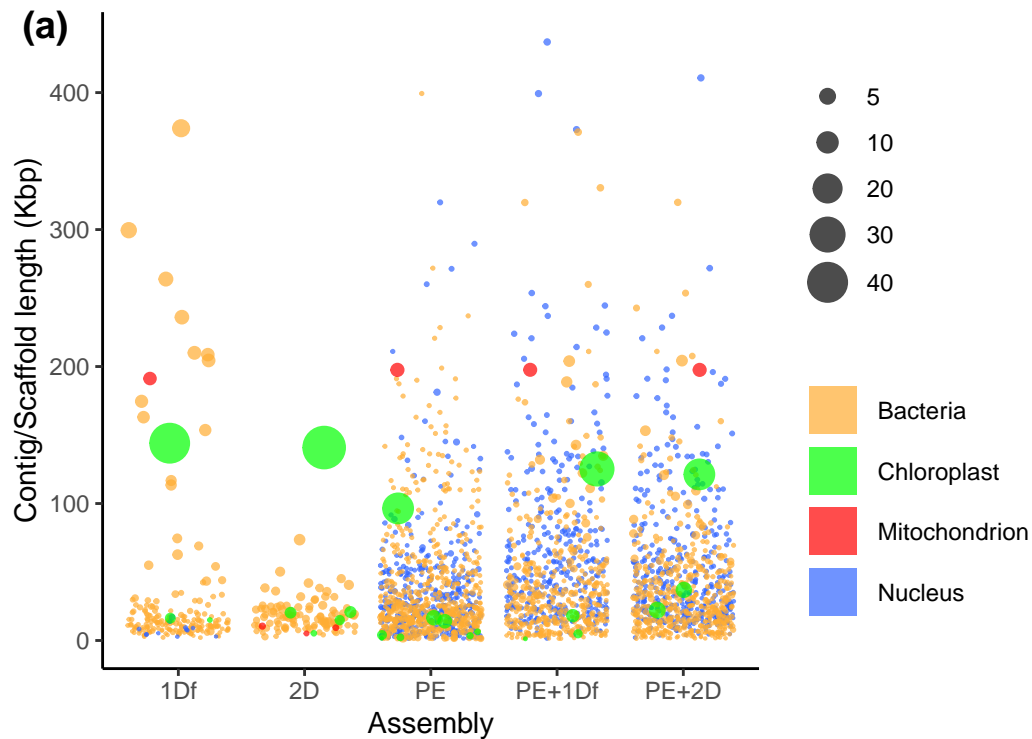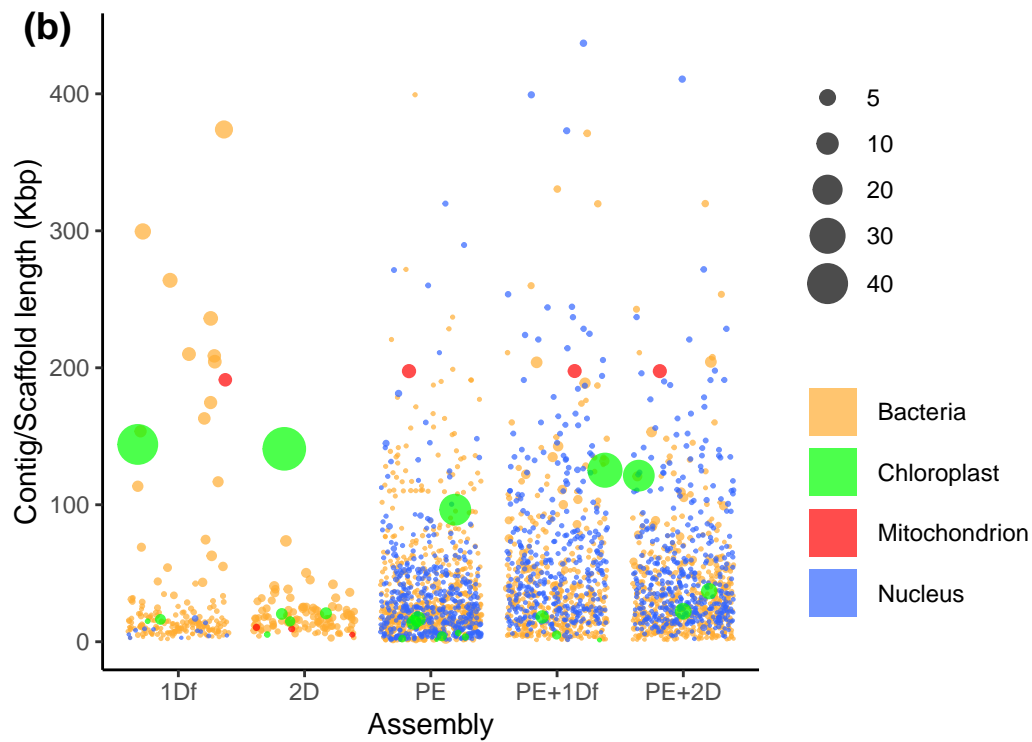

Supplement: Supplementary file 4 — Additional file 4: Figure S2. Assembly file content. Contig/scaffold abundance per genomic compartment across nanopore and hybrid assemblies. Bubble size represent the percentage of uniquely mapped reads to a given assembly and corresponding dataset (those representing < 1% no shown for figure clarity). Note that due to layering of the plot, numerous scaffolds < 20,000 bp in the Illumina and hybrid assemblies become hidden, thus two plots are shown to emphasize (a) bacterial scaffolds or (b) nuclear scaffolds. [file 12864_2019_6248_MOESM4_ESM.pdf]

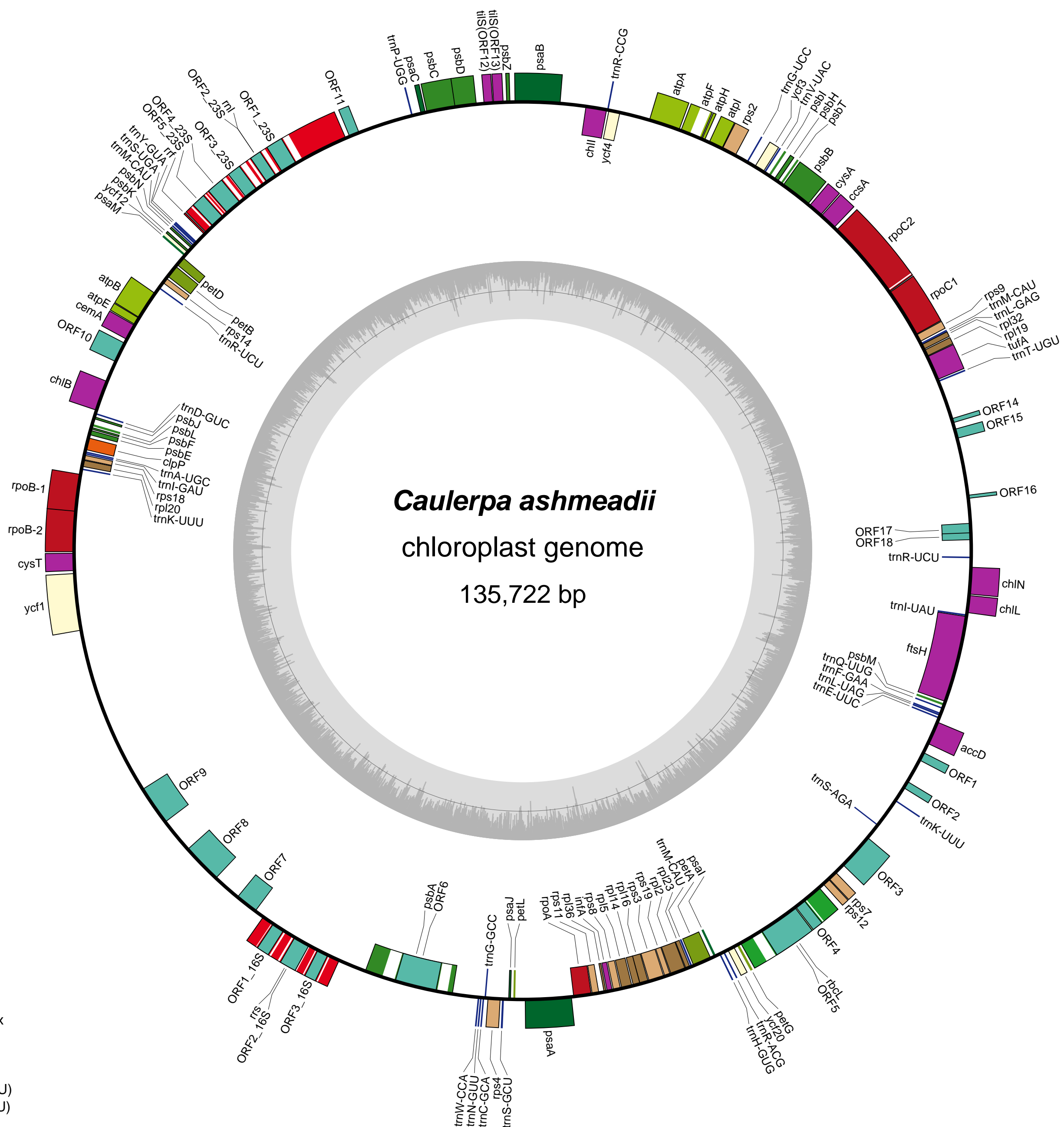

Supplement: Supplementary file 5 — Additional file 5: Figure S3. Circular Chloroplast genome map. [file 12864_2019_6248_MOESM5_ESM.pdf]

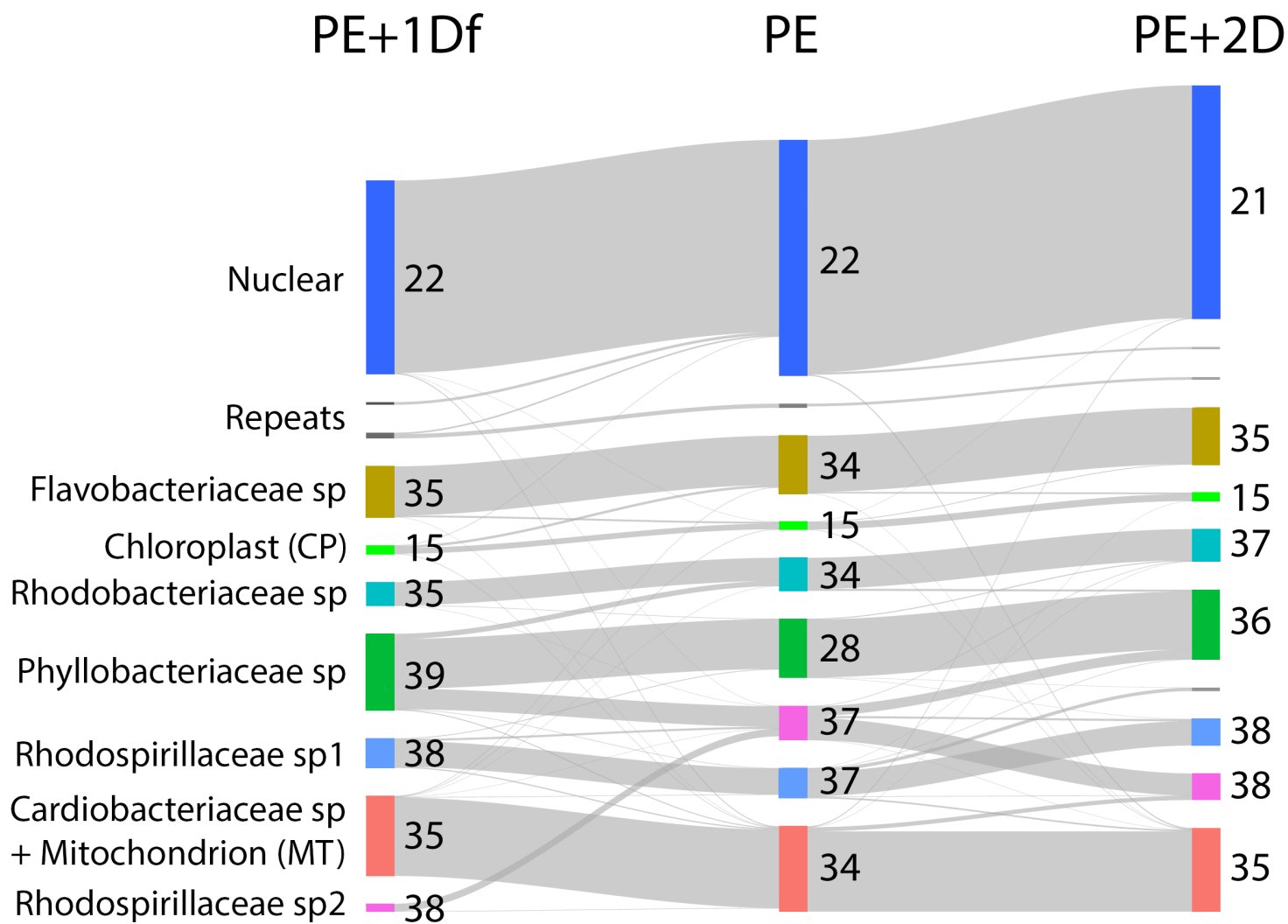

Supplement: Supplementary file 8 — Additional file 8: Figure S6. Bin reciprocity. Sankey plot depicting MyCC bin correspondence across Illumina and hybrid assembly files (Illumina PE + 1Df or 2D) and their taxonomy/origin in the Caulerpa ashmeadii holobiont based on COGs. Flow size linking bins represent the number of common scaffolds in the assembly files compared via reciprocal BLASTn. Numbers adjacent to bins represent the number of COGs reported by MyCC. Note the consistent delimitation of nuclear contigs but some instability in the binning of the Phyllobacteriaceae sp. and Rhodospirillaceae sp2. scaffolds. The Sankey diagram was built on the basis of html code available from Google Charts at https://developers.google.com/chart/interactive/docs/gallery/sankey. PE = Illumina Paired-Ends scaffolds, PE+1Df = hybrid 1Df scaffolds, PE+2D = hybrid 2D scaffolds. [file 12864_2019_6248_MOESM8_ESM.pdf]

(a)

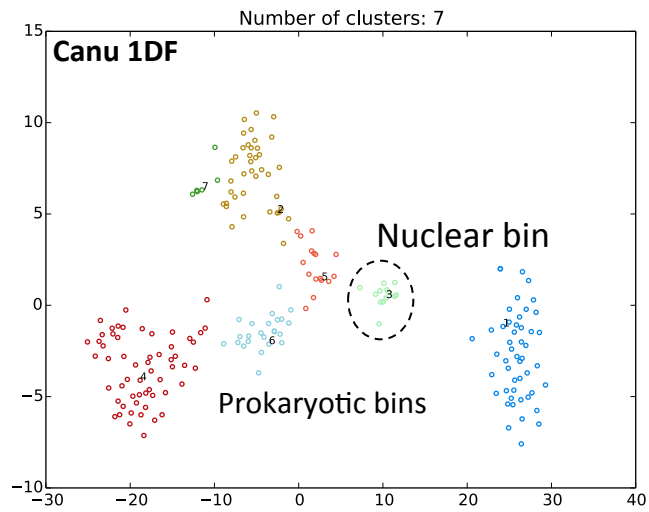

(b)

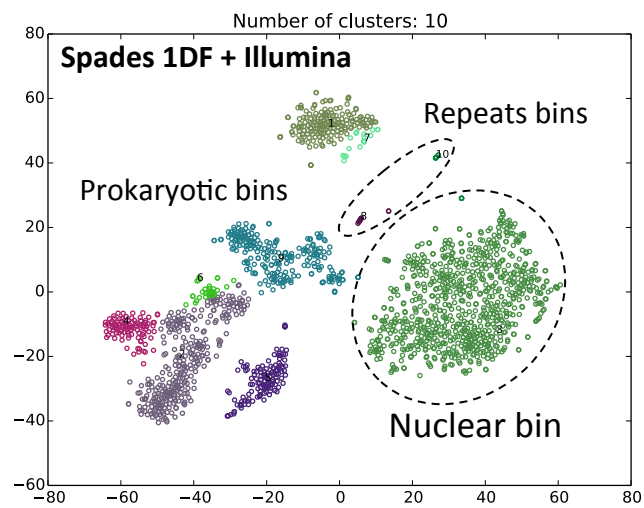

(c)

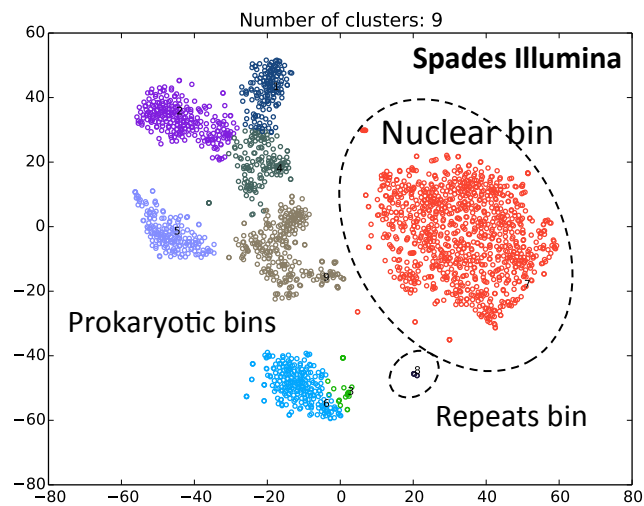

Supplement: Supplementary file 9 — Additional file 9: Figure S7. Metagenome binning. Comparison of delimited bins with MyCC (4mer) for three assemblies. (a) Experimental binning of Canu 1Df contigs. (b) Binning of Spades’ hybrid Illumina+1Df assembly. (c) Binning of Spades’ Illumina-only assembly. Bins containing nuclear and repeat contigs are emphasized with dashed circles. Remaining bins correspond to bacterial taxa. Note that printed bin numbers are unrelated between plots. [file 12864_2019_6248_MOESM9_ESM.pdf]

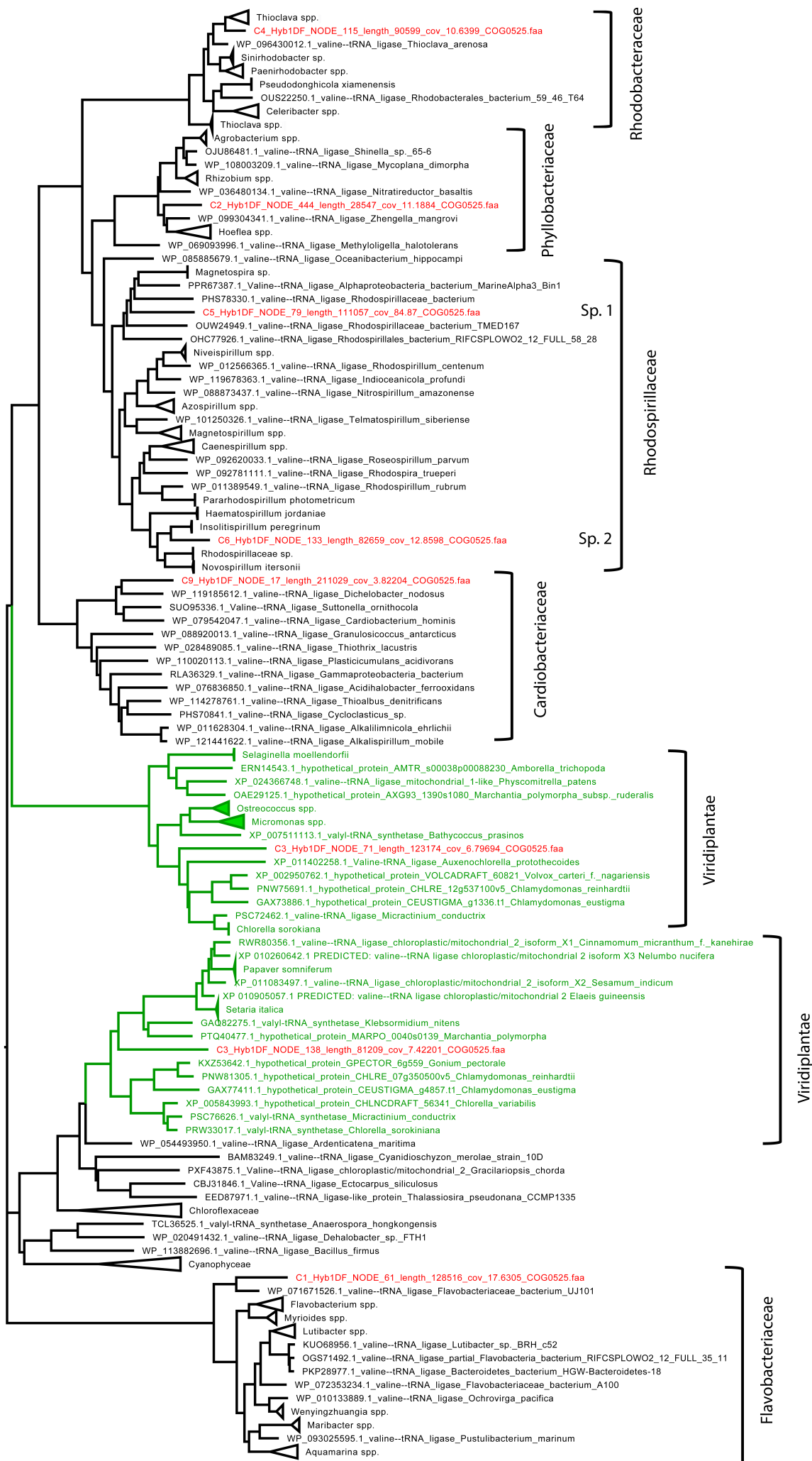

Supplement: Supplementary file 10 — Additional file 10: Figure S8. Phylogeny of valyl-tRNA ligase. Exploratory phylogeny of valyl-tRNA ligase (COG0525) amino acid sequences extracted from nuclear and bacterial bins by MyCC. For taxonomic identification of bins, sequence context was retrieved via BLASTp against GenBank’s non-redundant protein databases. Note the occurrence of two different valyl-tRNA ligases in Caulerpa ashmeadii nuclear genome. [file 12864_2019_6248_MOESM10_ESM.pdf]

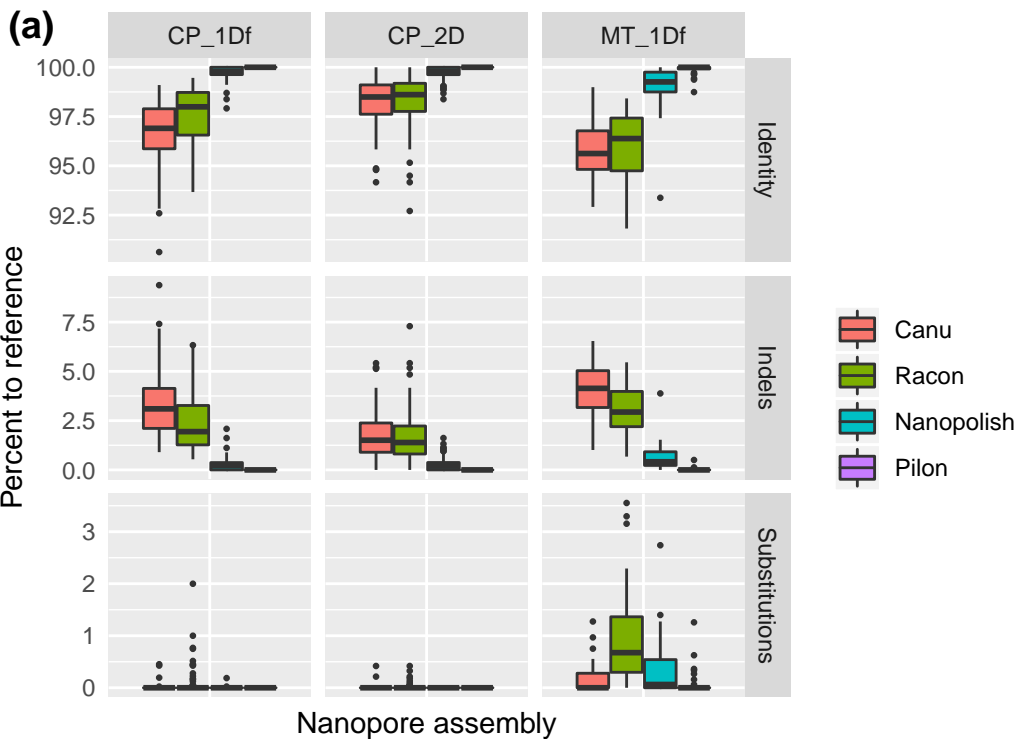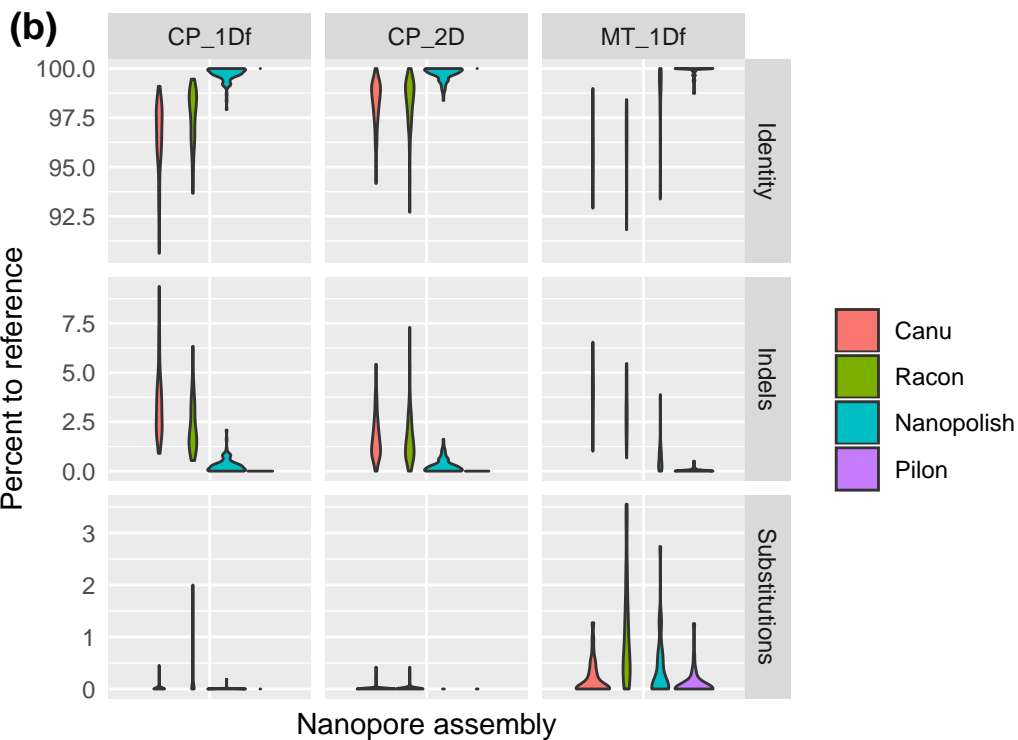

Supplement: Supplementary file 11 — Additional file 11: Figure S9. Polished quality of protein-encoding gene. Detailed distributions of gene quality following polishing steps as (a) boxplot or (b) violin plot. For comparison, numbers reported between parenthesis in Table 4 correspond to the cumulative percentage identity, indels and substitutions for all genes, while the data points and distribution in the present figure represent individual gene values. Note the perfect quality of chloroplast genes following Pilon polishing. CP_1DF=Chloroplast genes from 1Df assembly, CP_2D = Chloroplast genes from 2D assembly, MT_1Df = Mitochondrion genes from 1Df assembly. [file 12864_2019_6248_MOESM11_ESM.pdf]

### (a) Interspersed repeat (ASH1.2 versus ASH1.1)

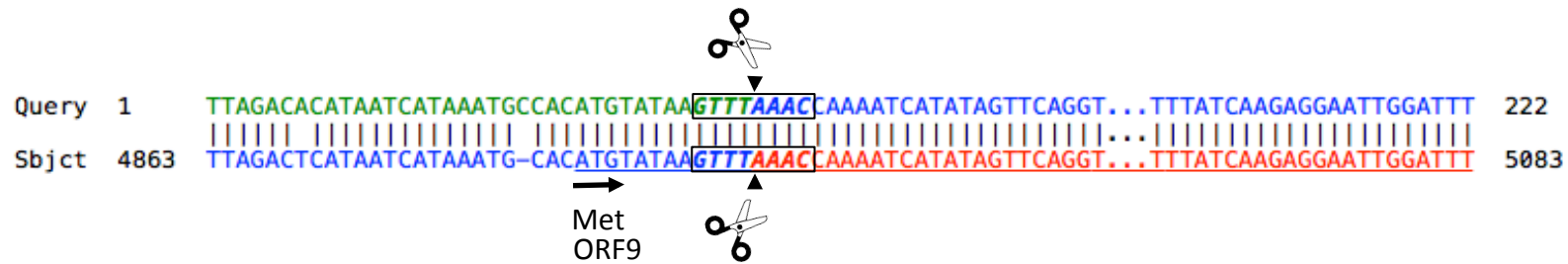

### (b) Recombined repeat (ASH1.3)

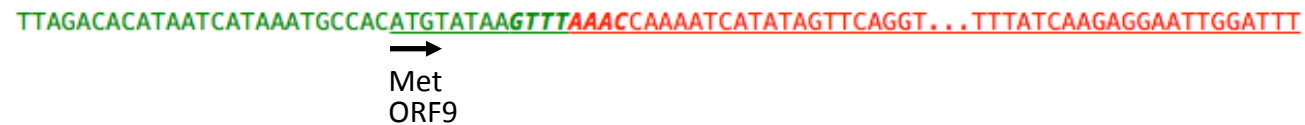

Supplement: Supplementary file 13 — Additional file 13: Figure S11. Putative chloroplast recombination mechanism. (a) Edited BLASTn report showing identity and common directionality of the interspersed repeats ASH1.1 and ASH1.2 and palindrome GTTTAAAC (italicized and boxed) acting as a potential endonuclease restriction site. Color coding represents boundaries of the putative excised (blue) and recombined genomic segments (green and red). Note that the blue fragment belonging to ASH1.2 on top extends up to the red fragment on the bottom on a physical distance of > 4600 bp up to ASH1.1 and ORF9 and exhibit the same directionality (i.e. see coordinates value) (b) Putative recombined repeat ASH1.3 at the palindromic site (green and red). Note that following recombination, the start codon and thus translation frame of ORF9 (Met for Methionine) is unaffected. [file 12864_2019_6248_MOESM13_ESM.pdf]

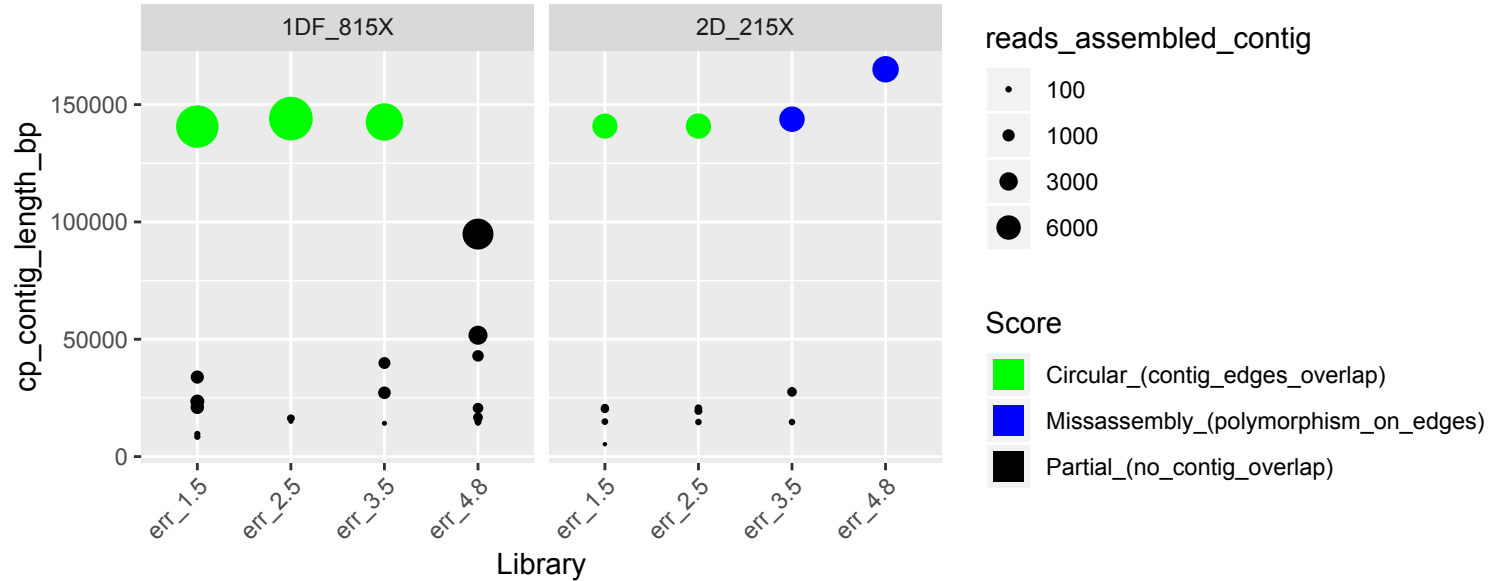

Supplement: Supplementary file 14 — Additional file 14: Figure S12. Error rate vs. assembly. Impact of Canu’s error rate parameter on assembly of the circular chloroplast genome in the presence of structural variation (SV) (black bubbles). Note the occurrence of misassemblies (i.e. polymorphism/SVs stitched on the contig’s extremities) when using relaxed error rates with 2D data, while 1D assembly still produces a circularizable contig (i.e. no misassembly). [file 12864_2019_6248_MOESM14_ESM.pdf]

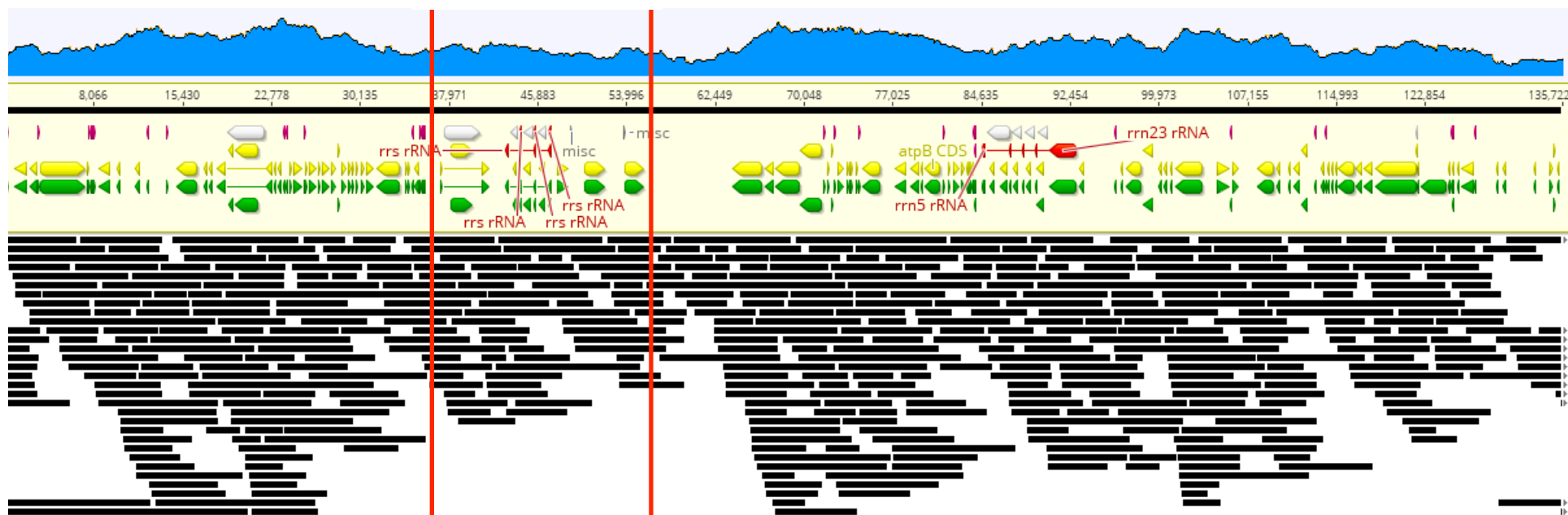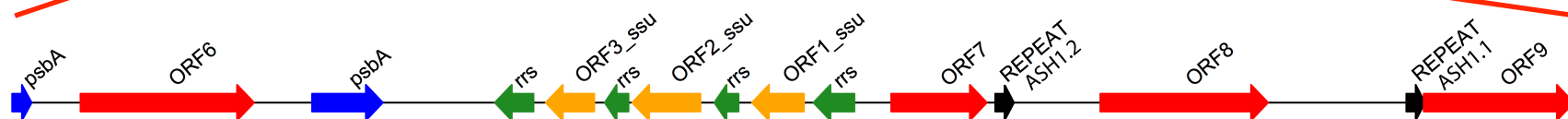

Supplement: Supplementary file 15 — Additional file 15: Figure S13. Chloroplast genome long reads’ pile-up. [file 12864_2019_6248_MOESM15_ESM.pdf]
