# Supplementary material for: Promising prospects of nanopore sequencing for algal hologenomics and structural variation discovery
Source: BMC Genomics. 2019 Nov 13;20:850. doi: 10.1186/s12864-019-6248-2 (PMC6854639; doi:10.1186/s12864-019-6248-2)
Supplement: Supplementary file 6 — Additional file 6: Figure S4. Circular Mitochondrion genome map. [file 12864_2019_6248_MOESM6_ESM.pdf]

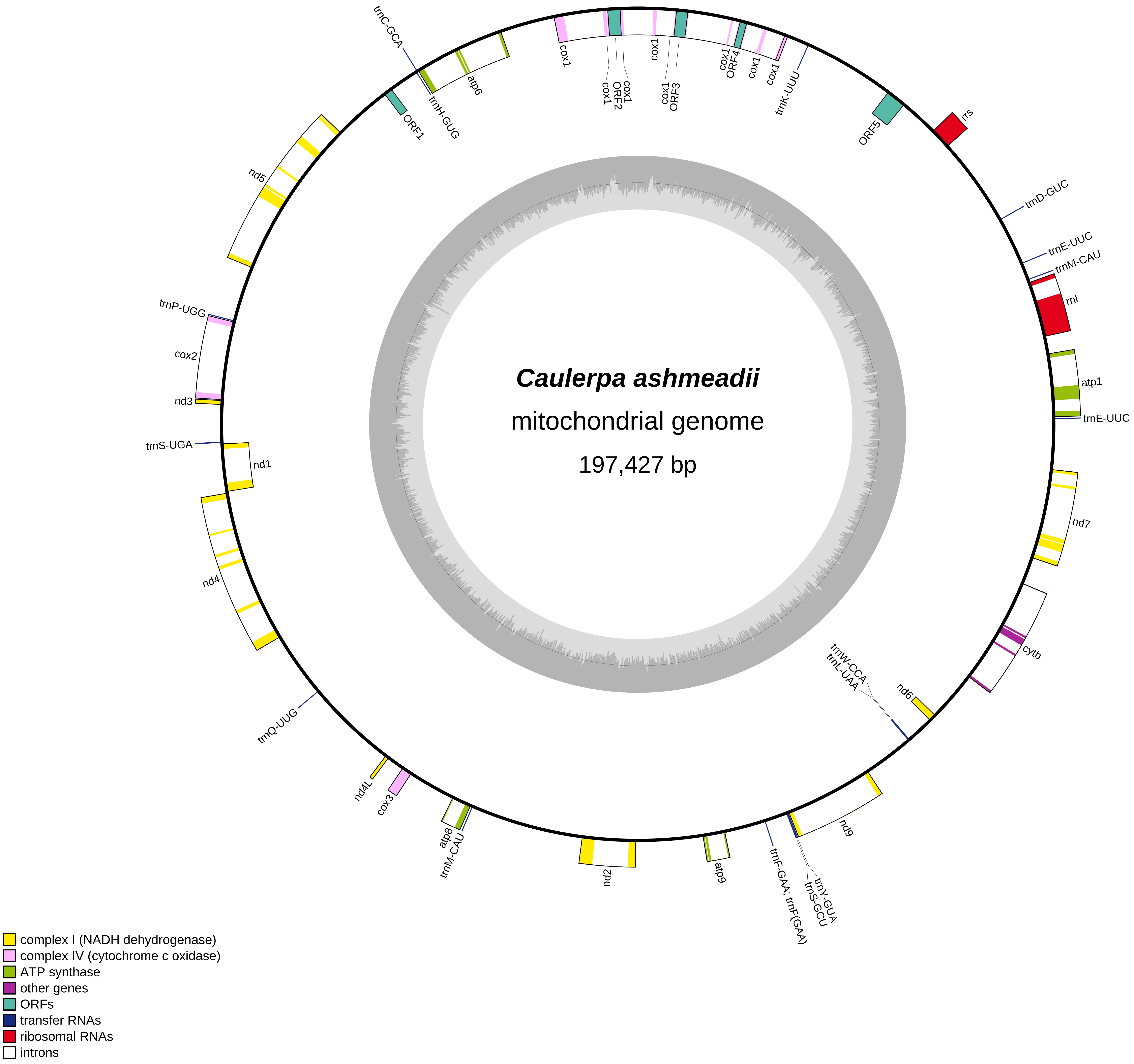
Caulerpa ashmeadii
mitochondrial genome
197,427 bp
cox1
cox1
ORF2
cox1
cox1
cox1
ORF3
cox1
ORF4
cox1
cox1
trnK-UUU
ORF5
rrs
trnD-GUC
trnE-UUC
trnM-CAU
rnl
atp1
trnE-UUC
nd7
cytb
nd6
trnW-CCA
trnL-UAA
nd9
trnY-GUA
trnS-GCU
trnF-GAA; trnF(GAA)
atp9
nd2
trnM-CAU
atp8
cox3
nd4L
trnQ-UUG
nd4
nd1
trnS-UGA
nd3
cox2
trnP-UGG
nd5
ORF1
trnH-GUG
atp6
trnC-GCA
complex I (NADH dehydrogenase)
complex IV (cytochrome c oxidase)
ATP synthase
other genes
ORFs
transfer RNAs
ribosomal RNAs
introns
