# Supplementary material for: Promising prospects of nanopore sequencing for algal hologenomics and structural variation discovery
Source: BMC Genomics. 2019 Nov 13;20:850. doi: 10.1186/s12864-019-6248-2 (PMC6854639; doi:10.1186/s12864-019-6248-2)
Supplement: Supplementary file 7 — Additional file 7: Figure S5. GenomeScope profile. Kmer coverage and log coverage for the entire Illumina dataset (a and b), the dataset depleted of bacterial reads (c and d), and the dataset depleted of nuclear reads (e and f). Note the drop in kmer frequencies indicated by arrows and circles to point at the depletion of bacterial kmers overlapping with nuclear ones (a and c) and their recovery in the dataset depleted of nuclear reads (e). Note as well the successful depletion of the main bacterial kmer peak (*) in the bacterial depleted dataset (a and e vs. c). [file 12864_2019_6248_MOESM7_ESM.pdf]

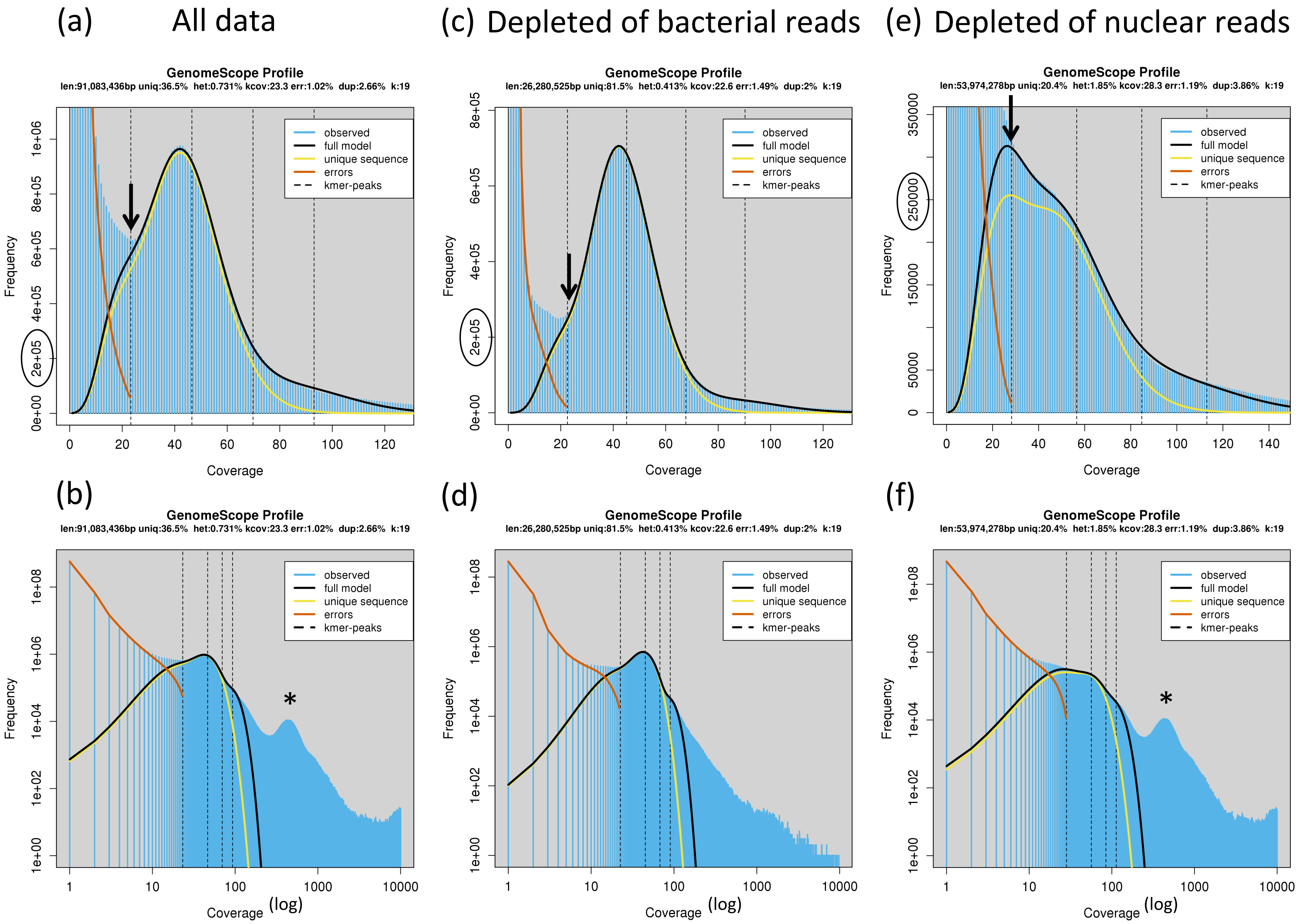
(a) All data
(c) Depleted of bacterial reads
(e) Depleted of nuclear reads
(b)
(d)
(f)
GenomeScope Profile
len:91,083,436bp uniq:36.5% het:0.731% kcov:23.3 err:1.02% dup:2.66% k:19
len:26,280,525bp uniq:81.5% het:0.413% kcov:22.6 err:1.49% dup:2% k:19
len:53,974,278bp uniq:20.4% het:1.85% kcov:28.3 err:1.19% dup:3.86% k:19
observed
full model
unique sequence
errors
kmer-peaks
Frequency
Coverage
Coverage (log)
*

GenomeScope version 1.0
k = 19

property	min	max
Heterozygosity	0.409485%	0.416116%
Genome Haploid Length	26,268,957 bp	26,280,525 bp
Genome Repeat Length	4,867,943 bp	4,870,086 bp
Genome Unique Length	21,401,014 bp	21,410,439 bp
Model Fit	97.5292%	99.2466%
Read Error Rate	1.49446%	1.49446%
