# Supplementary material for: Promising prospects of nanopore sequencing for algal hologenomics and structural variation discovery
Source: BMC Genomics. 2019 Nov 13;20:850. doi: 10.1186/s12864-019-6248-2 (PMC6854639; doi:10.1186/s12864-019-6248-2)
Supplement: Supplementary file 12 — Additional file 12: Figure S10. BLASTn report for atp1/atpA. BLASTn alignment of mitochondrial atp1 and chloroplast atpA. Note identity level nearing ~ 70%, causing mismapping of low quality atpA reads on atp1. [file 12864_2019_6248_MOESM12_ESM.pdf]

```
BLASTN 2.9.0+
Reference: Stephen F. Altschul, Thomas L. Madden, Alejandro
A. Schaffer, Jinghui Zhang, Zheng Zhang, Webb Miller, and
David J. Lipman (1997), "Gapped BLAST and PSI-BLAST: a new
generation of protein database search programs", Nucleic
Acids Res. 25:3389-3402.

RID: 9AC73PMW114

Query= atp1

Length=864
                                                                     Score        E     Max
Sequences producing significant alignments:                         (Bits)     Value  ident

Query_75155 atpA                                                     343        4e-98  69%

ALIGNMENTS
>Query_75155 atpA
Length=1533

 Score = 343 bits (380),  Expect = 4e-98
 Identities = 578/832 (69%), Gaps = 26/832 (3%)
 Strand=Plus/Plus

Query  40    GCCCCCGGGATTATTGCTCGCCGTTCTGTACACGAGCCGATGCAAACTGGAATTAAAGCT  99
             || || || |||||||| || |  |||||    || ||  | ||||| ||  ||  ||||
Sbjct  403   GCACCTGGTATTATTGCACGACAATCTGTTTTTGAACCATTACAAACAGGGCTTGTAGCT  462

Query  100   GTGGATAGTCTGGTTCCCATCGGCCGTGGTCAACGGGAATTAATTATTGGGGATCGTCAA  159
              | |||  | || |||| || ||  | || ||||| |||||||||||||| ||| | |||
Sbjct  463   ATCGATTCTATGATTCCTATAGGTAGAGGGCAACGAGAATTAATTATTGGTGATAGACAA  522

Query  160   ACCGGTAAAACAGCAATCGCTTTGGATACTATTTTGAATCAAAAACACAACACTGGAGAG  219
             || || |||||| |||||||| | |||||||||||||||||||||   ||  |||
Sbjct  523   ACAGGAAAAACATCAATCGCTGTTGATACTATTTTGAATCAAAAAGGTAAATCTG-----  577

Query  220   GCTCAGCTCTATTGTGTCTATGTGGCCATTGGTCAAAAACGGTCAACCGTGGCCCA-GTT  278
                    | |||||  | ||||| || ||||| ||||||   ||  |  | || || |||
Sbjct  578   -------TTTATTGCATTTATGTAGCTATTGGACAAAAAGCTTCCTCTATTGCTCAAGTT  630

Query  279   GGTTAAAATTTTATCTGACGCTCAGGCAATGGAGTATACCACTGTAGTGGCTGCCACGGC  338
             | ||  | ||| |    ||     ||| ||||| ||||| | | | || |||||  | ||
Sbjct  631   GTTTCGACTTTAAAGAAACAAGG-GGCTATGGAATATACTATTATTGTTGCTGCACCAGC  689

Query  339   CTCAGAT---CCCGCGCCTTTACAGTTTTTAGCACCTTATACTGGATGTGCTATATCTGA  395
                ||||   || ||  ||||||| | |||||| |||||||||||   | || |  | ||
Sbjct  690   ---AGATTCTCCAGCTACTTTACAATATTTAGCTCCTTATACTGGTGCTACTTTGGCAGA  746

Query  396   ATTTTTCCGGGATAATGGTATGCATGCGCTCATTATTTTCGATGATCTCAGCAAACAAAG  455
             || ||||  | |||| ||    ||| | ||  | ||||  |||||| |    ||||||
Sbjct  747   ATATTTCATGTATAAAGGAGGTCATACCCTTGTGATTTATGATGATTTATCTAAACAAGC  806

Query  456   TGTGGCCTATAGGCAAATGTCACTATTGTTGCGTCGACCTCCCGGTAGAGAGGCGTTTCC  515
             |   || |||||  |||||||  || | ||  | ||||| || ||| | || || |||||
Sbjct  807   TCAAGCTTATAGAGAAATGTCTTTACTTTTAAGACGACCACCTGGTCGTGAAGCTTTTCC  866

Query  516   AGGAGATGTATTTTATCTTCATAGTCGACTACTTGAACGGGCTGCGAAACTAAGTGATAC  575
              || ||||| ||||||||||||    ||||  | |||||||| || ||||| | | |
Sbjct  867   TGGGGATGTTTTTTATCTTCATTCAAGACTTTTAGAACGGGCAGCAAAACTGAATAAACA  926

Query  576   ACTAGGAGCAGGCAGTATGACCGCCTTACCTATCATTGAAACACAATCGGGGGATGTGTC  635
             | | || | ||| |||||||| ||  | |||||  |||||||||||   || ||||| ||
Sbjct  927   AATGGGTGGAGGTAGTATGACAGCTCTTCCTATTGTTGAAACACAAGAAGGAGATGTATC  986

Query  636   GGCTTATATTCCTACCAATGTGATTTCGATTACTGATGGACAAATATTCCTGGAAACATC  695
              ||||||||||| || ||||| ||||| |||||||||||||| || ||  |   | || |
Sbjct  987   TGCTTATATTCCAACAAATGTAATTTCTATTACTGATGGACAGATTTTTT---ATCAGC  1043

Query  696   A-CTATTTT--ATAAAGGTATTCGTCCAGCAATTAATGTGGGAATTTCTGTGAGTCGTGT  752
             |  ||||||  |||  || ||||||||||| |||||||| || ||||||||   ||||||
Sbjct  1044  AGATATTTTTAATAGTGGAATTCGTCCAGCTATTAATGTTGGTATTTCTGTTTCTCGTGT  1103
```

```
Query  753   GGGTTCAGCCGCACAATTAAAAGCAATGAAACAGGTGGCCGGAAGTTTAAAATTGGAATT  812
              || || || || |||  |||||| |||||| | ||||| ||    |||||||| |||||
Sbjct  1104  AGGGTCGGCTGCTCAACCAAAAGCTATGAAAAAAGTGGCTGGTCAATTAAAATTAGAATT  1163

Query  813   AGCTCAGTATAGAGAGGTCGCAGCGTTTGCACAATTTGGTTCTGACTTAGAT  864
             |||||| | |   ||  | | ||| ||| |||||||   ||| || ||||||
Sbjct  1164  AGCTCAATTTGCTGAACTTGAAGCTTTTTCACAATTCTCTTCAGATTTAGAT  1215
```
