# Supplementary material for: Promising prospects of nanopore sequencing for algal hologenomics and structural variation discovery
Source: BMC Genomics. 2019 Nov 13;20:850. doi: 10.1186/s12864-019-6248-2 (PMC6854639; doi:10.1186/s12864-019-6248-2)
Supplement: Supplementary file 16 — Additional file 16: Figure S14. BLASTp report for psbA intronic ORFs. [file 12864_2019_6248_MOESM16_ESM.pdf]

BLASTP 2.10.0+
Reference: Stephen F. Altschul, Thomas L. Madden, Alejandro
A. Schaffer, Jinghui Zhang, Zheng Zhang, Webb Miller, and
David J. Lipman (1997), "Gapped BLAST and PSI-BLAST: a new
generation of protein database search programs", Nucleic
Acids Res. 25:3389-3402.

Reference for compositional score matrix adjustment: Stephen
F. Altschul, John C. Wootton, E. Michael Gertz, Richa
Agarwala, Aleksandr Morgulis, Alejandro A. Schaffer, and
Yi-Kuo Yu (2005) "Protein database searches using
compositionally adjusted substitution matrices", FEBS J.
272:5101-5109.

RID: SA1045XE01R

Database: All non-redundant GenBank CDS
translations+PDB+SwissProt+PIR+PRF excluding environmental samples
from WGS projects
234,979,574 sequences; 84,697,594,569 total letters

############

Query= ORF6

Length=683

Sequences producing significant alignments:	Score (Bits)	E Value	Max Ident
YP_009519247.1 hypothetical protein [Caulerpa verticillata]	1040	0.0	73%
AOP19042.1 hypothetical protein [Halimeda discoidea]	873	0.0	61%
YP_009514393.1 hypothetical protein [Caulerpa lentillifera]	378	2e-123	64%
ARR28427.1 hypothetical protein [Caulerpa okamurae]	376	7e-123	65%
ALO20849.1 HNH endonuclease [Chlamydomonas nivalis]	366	2e-113	35%
YP_009519478.1 hypothetical protein [Rhipiliopsis peltata]	350	2e-107	37%
YP_009184986.1 putative reverse transcriptase, maturase and HN...	339	2e-102	34%
ALO20843.1 hypothetical protein [Chlamydomonas nivalis]	328	6e-100	37%
AWI68212.1 hypothetical protein [Pediastrum angulosum]	332	8e-100	34%
AOC61484.1 putative reverse transcriptase and intron maturase ...	323	8e-98	33%
WP_136982224.1 nicotine oxidoreductase [Vibrio sp. F13]	321	6e-97	34%
QDR25075.1 putative reverse transcriptase and intron maturase ...	320	1e-96	35%
YP_009472852.1 hypothetical protein [Caulerpa manorensis]	319	3e-96	34%
WP_042082597.1 hypothetical protein [Aeromonas veronii]	317	2e-95	34%
WP_113736215.1 MULTISPECIES: hypothetical protein [Aeromonas]	315	9e-95	34%
WP_144502814.1 group II intron reverse transcriptase/maturase ...	315	1e-94	36%
WP_003459374.1 nicotine oxidoreductase [Pseudomonas aeruginosa]	315	1e-94	35%
WP_130241175.1 nicotine oxidoreductase [Vibrio vulnificus]	315	1e-94	34%
AYE93200.1 hypothetical protein C0995_000022 [Termitomyces sp.]	318	1e-94	35%
WP_087983616.1 group II intron reverse transcriptase/maturase ...	314	2e-94	36%
YP_009184874.1 putative reverse transcriptase, intron maturase...	313	5e-94	35%
WP_104323339.1 nicotine oxidoreductase [Marinobacter sp. N4]	313	8e-94	33%
WP_078557596.1 hypothetical protein [Vibrio parahaemolyticus]	313	8e-94	33%
WP_124886972.1 nicotine oxidoreductase [Burkholderia stagnalis]	313	9e-94	32%
WP_085917292.1 nicotine oxidoreductase [Halomonas sp. CSM-2]	312	1e-93	34%
WP_113624435.1 MULTISPECIES: nicotine oxidoreductase [Vibrio]	312	1e-93	33%
WP_017684078.1 hypothetical protein [Pseudomonas syringae]	312	1e-93	35%
WP_017703896.1 hypothetical protein [Pseudomonas syringae]	312	1e-93	35%
WP_085714244.1 hypothetical protein [Pseudomonas syringae]	311	2e-93	35%
WP_103653046.1 group II intron reverse transcriptase/maturase ...	310	5e-93	36%
WP_044046873.1 nicotine oxidoreductase [Pseudomonas putida]	310	1e-92	34%
YP_009367524.1 putative reverse transcriptase and intron matur...	310	1e-92	33%
WP_098472746.1 group II intron reverse transcriptase/maturase ...	309	1e-92	36%
WP_095037225.1 MULTISPECIES: nicotine oxidoreductase [Pseudomo...	309	2e-92	33%
WP_021728157.1 MULTISPECIES: Reverse transcriptase / RNA matur...	309	2e-92	36%
AMX22269.1 hypothetical protein [Cryphonectria parasitica]	312	6e-92	34%
ATI20475.1 group II intron reverse transcriptase/maturase [Jug...	312	8e-92	34%
WP_076775706.1 group II intron reverse transcriptase/maturase ...	307	8e-92	35%
AEJ10966.1 nicotine oxidoreductase [Pseudomonas putida S16]	309	9e-92	34%
TMD92013.1 RNA-dependent DNA polymerase [Chloroflexi bacterium]	307	1e-91	32%
WP_016078729.1 hypothetical protein [Bacillus cereus]	307	1e-91	36%
KHD04731.1 hypothetical protein PN36_19900 [Candidatus Thiomar...	307	1e-91	33%
CAA38781.1 reverse transcriptase homologue COI iA grp II prote...	311	1e-91	34%
TMC24583.1 RNA-dependent DNA polymerase [Chloroflexi bacterium]	306	2e-91	32%
WP_114292142.1 hypothetical protein [Sulfitobacter sp. PIC-76]	307	2e-91	33%
AMX22256.1 hypothetical protein [Cryphonectria parasitica]	311	2e-91	33%
WP_106082431.1 group II intron reverse transcriptase/maturase ...	305	3e-91	36%
WP_001149933.1 MULTISPECIES: maturase [Bacillus cereus group]	305	3e-91	36%
AKO77651.1 hypothetical protein EN12_21235 [Vibrio cholerae]	306	4e-91	32%
WP_016090496.1 hypothetical protein [Bacillus cereus]	305	4e-91	35%
WP_099686794.1 group II intron reverse transcriptase/maturase ...	305	5e-91	36%
WP_144533802.1 group II intron reverse transcriptase/maturase ...	305	5e-91	36%
WP_114134779.1 hypothetical protein [Cupriavidus necator]	305	7e-91	31%

WP_001149934.1 MULTISPECIES: maturase [Bacillus cereus group]	304	1e-90	35%
WP_007922378.1 RNA-directed DNA polymerase [Ktedonobacter race...	304	1e-90	33%
WP_098537552.1 group II intron reverse transcriptase/maturase ...	304	1e-90	35%
TMD28872.1 RNA-dependent DNA polymerase [Chloroflexi bacterium]	304	1e-90	33%
WP_097920007.1 group II intron reverse transcriptase/maturase ...	304	1e-90	35%
WP_048561943.1 maturase [Bacillus cereus]	303	2e-90	35%
WP_001149935.1 MULTISPECIES: maturase [Bacillus cereus group]	303	3e-90	35%
WP_044798328.1 MULTISPECIES: maturase [Bacillus]	303	3e-90	35%
WP_129893139.1 RNA-dependent DNA polymerase [Ktedonobacterales...	305	3e-90	32%
WP_114946279.1 hypothetical protein [Microvirga sp. CCBAU 65841]	303	3e-90	33%
WP_089608005.1 group II intron reverse transcriptase/maturase ...	302	5e-90	35%
WP_098442215.1 group II intron reverse transcriptase/maturase ...	302	5e-90	35%
WP_033698193.1 maturase [Bacillus thuringiensis]	302	6e-90	35%
WP_016099972.1 hypothetical protein [Bacillus cereus]	302	7e-90	35%
WP_044157303.1 maturase [Bacillus thuringiensis]	302	9e-90	35%
SFF27114.1 group II intron reverse transcriptase/maturase [Aci...	302	9e-90	32%
GBH18395.1 Retron-type reverse transcriptase [Pseudomonas syri...	301	9e-90	35%
WP_007914005.1 RNA-directed DNA polymerase [Ktedonobacter race...	302	1e-89	33%
WP_092941870.1 hypothetical protein [Acidovorax wautersii]	301	1e-89	32%
WP_119684256.1 group II intron reverse transcriptase/maturase ...	301	1e-89	35%
YP_009492182.1 hypothetical protein [Pseudopediastrum integrum]	303	1e-89	31%
WP_074644204.1 MULTISPECIES: group II intron reverse transcrip...	301	2e-89	35%
WP_016097415.1 hypothetical protein [Bacillus cereus]	301	2e-89	35%
WP_098344211.1 group II intron reverse transcriptase/maturase ...	301	2e-89	35%
WP_007921601.1 RNA-directed DNA polymerase [Ktedonobacter race...	301	2e-89	33%
WP_007914435.1 RNA-directed DNA polymerase [Ktedonobacter race...	301	2e-89	33%
WP_007921348.1 RNA-directed DNA polymerase [Ktedonobacter race...	301	3e-89	33%
WP_087947829.1 group II intron reverse transcriptase/maturase ...	300	4e-89	35%
WP_119782079.1 group II intron reverse transcriptase/maturase ...	300	4e-89	35%
WP_128267365.1 group II intron reverse transcriptase/maturase ...	300	4e-89	35%
AAA32006.2 cytochrome c oxidase subunit I [Podospora anserina]	305	5e-89	33%
TMC21115.1 RNA-dependent DNA polymerase [Chloroflexi bacterium]	300	7e-89	32%
OZO38125.1 hypothetical protein CGU41_19175 [Pseudomonas aerug...	299	2e-88	33%
YP_009550009.1 Reverse transcriptase COI ialpha grpII protein ...	303	2e-88	34%
WP_125908501.1 group II intron reverse transcriptase/maturase ...	298	2e-88	33%
WP_098156398.1 MULTISPECIES: group II intron reverse transcrip...	298	2e-88	35%
YP_009420548.1 hypothetical protein [Ulva pertusa]	300	2e-88	34%
WP_038663527.1 nicotine oxidoreductase [Pseudomonas stutzeri]	298	3e-88	33%
WP_088338955.1 group II intron reverse transcriptase/maturase ...	298	3e-88	34%
QDP17704.1 reverse transcriptase domain-containing protein [Sc...	303	3e-88	33%
TXI28265.1 hypothetical protein E6Q69_16610 [Pseudomonas alcal...	298	3e-88	33%
WP_123634498.1 hypothetical protein [Marinobacter sp. R17]	297	5e-88	33%
WP_060567105.1 nicotine oxidoreductase [Massilia sp. WG5]	297	7e-88	32%
WP_098099224.1 group II intron reverse transcriptase/maturase ...	295	2e-87	35%
WP_106103073.1 group II intron reverse transcriptase/maturase ...	295	2e-87	35%
YP_002600927.1 putative reverse transcriptase and intron matur...	295	2e-87	33%
SCI19716.1 Group II intron-encoded protein ltrA [uncultured Ru...	295	2e-87	32%

############

Query= ORF20

Length=603

Sequences producing significant alignments:	Score (Bits)	E Value	Max Ident
YP_009185223.1 putative reverse transcriptase, intron maturase...	430	6e-140	39%
YP_636002.1 intron-encoded reverse transcriptase [Tetradesmus ...	418	1e-135	39%
ASY96128.1 Group II intron-encoded protein LtrA [Tetradesmus o...	407	4e-131	38%
P19593.1 RecName: Full=Probable reverse transcriptase [Tetrade...	392	1e-125	37%
YP_009519136.1 hypothetical protein [Caulerpa verticillata]	346	1e-113	97%
YP_009463644.1 RNA-dependent DNA polymerase [Haematococcus lac...	313	7e-97	39%
ALO21699.1 putative HNH endonuclease [Stephanosphaera pluvialis]	288	7e-86	35%
YP_009326792.1 hypothetical protein [Caulerpa racemosa]	254	1e-72	33%
AKF33562.1 hypothetical protein [Ulva sp. UNA00071828]	249	4e-71	31%
YP_009519201.1 hypothetical protein [Caulerpa verticillata]	236	8e-71	65%
YP_009519135.1 hypothetical protein [Caulerpa verticillata]	229	5e-69	85%
ARR28461.1 hypothetical protein [Caulerpa okamurae]	241	9e-68	33%
AYE93292.1 DNA polymerase [Termitomyces sp.]	239	2e-67	31%
BAY52511.1 maturase reverse transcriptase [Thermosynechococcus...	233	3e-65	31%
WP_126987904.1 group II intron reverse transcriptase/maturase ...	233	4e-65	31%
YP_009519200.1 hypothetical protein [Caulerpa verticillata]	220	4e-65	66%
WP_134015154.1 group II intron reverse transcriptase/maturase ...	232	6e-65	30%
WP_045994405.1 group II intron reverse transcriptase/maturase ...	232	6e-65	30%
WP_049396991.1 group II intron reverse transcriptase/maturase ...	232	8e-65	32%
WP_109337812.1 group II intron reverse transcriptase/maturase ...	232	9e-65	32%
WP_107231717.1 MULTISPECIES: group II intron reverse transcrip...	231	1e-64	32%
WP_032957202.1 group II intron reverse transcriptase/maturase ...	231	2e-64	32%
WP_071229638.1 MULTISPECIES: group II intron reverse transcrip...	230	4e-64	31%
WP_020429325.1 group II intron reverse transcriptase/maturase ...	229	8e-64	31%
WP_061057251.1 group II intron reverse transcriptase/maturase ...	229	9e-64	32%
WP_011055886.1 group II intron reverse transcriptase/maturase ...	229	1e-63	31%
WP_143682674.1 group II intron reverse transcriptase/maturase ...	229	1e-63	31%
WP_011057003.1 group II intron reverse transcriptase/maturase ...	229	2e-63	31%
WP_011056164.1 group II intron reverse transcriptase/maturase ...	229	2e-63	31%
WP_011055969.1 group II intron reverse transcriptase/maturase ...	228	2e-63	31%

YP_009240571.1 RNA-dependent DNA polymerase [Pyronema omphalodes]	229	4e-63	30%
WP_061057252.1 group II intron reverse transcriptase/maturase ...	228	5e-63	32%
TQF64774.1 reverse transcriptase [Pyrrhoderma noxium]	228	6e-63	30%
WP_075988062.1 group II intron reverse transcriptase/maturase ...	227	7e-63	32%
WP_126984859.1 group II intron reverse transcriptase/maturase ...	226	1e-62	30%
WP_089681253.1 group II intron reverse transcriptase/maturase ...	225	1e-62	31%
BAY51406.1 reverse transcriptase [Thermosynechococcus vulcanus...	226	2e-62	30%
WP_087388264.1 group II intron reverse transcriptase/maturase ...	226	2e-62	34%
WP_117475412.1 group II intron reverse transcriptase/maturase ...	225	4e-62	34%
WP_122333818.1 group II intron reverse transcriptase/maturase ...	225	4e-62	34%
WP_007844350.1 MULTISPECIES: group II intron reverse transcrip...	224	4e-62	34%
AYE93236.1 hypothetical protein C0995_000069 [Termitomyces sp.]	225	5e-62	30%
WP_117899849.1 group II intron reverse transcriptase/maturase ...	224	1e-61	34%
WP_072677698.1 group II intron reverse transcriptase/maturase ...	224	1e-61	32%
ECA5087620.1 group II intron reverse transcriptase/maturase [S...	222	3e-61	31%
OJT01524.1 group II intron reverse transcriptase/maturase [Mar...	221	4e-61	32%
WP_040153628.1 group II intron reverse transcriptase/maturase ...	221	7e-61	29%
EAP5604862.1 group II intron reverse transcriptase/maturase [S...	221	9e-61	31%
WP_096036791.1 group II intron reverse transcriptase/maturase ...	219	1e-60	32%
NP_150378.1 hypothetical protein PylioMp02 [Pylaiella littoralis]	221	1e-60	31%
WP_102451446.1 group II intron reverse transcriptase/maturase ...	219	2e-60	32%
WP_001930107.1 group II intron reverse transcriptase/maturase ...	220	2e-60	31%
EBZ5772484.1 group II intron reverse transcriptase/maturase [S...	220	2e-60	31%
WP_027697944.1 group II intron reverse transcriptase/maturase ...	220	2e-60	31%
EBA4495834.1 group II intron reverse transcriptase/maturase [S...	220	3e-60	30%
WP_007462986.1 group II intron reverse transcriptase/maturase ...	220	3e-60	30%
WP_048899869.1 group II intron reverse transcriptase/maturase ...	219	4e-60	32%
WP_102559542.1 group II intron reverse transcriptase/maturase ...	218	4e-60	32%
EBG5206135.1 group II intron reverse transcriptase/maturase [S...	219	5e-60	30%
WP_001727958.1 group II intron reverse transcriptase/maturase ...	219	5e-60	31%
WP_065300429.1 group II intron reverse transcriptase/maturase ...	218	5e-60	31%
WP_107193931.1 group II intron reverse transcriptase/maturase ...	219	5e-60	31%
MIF48517.1 group II intron reverse transcriptase/maturase [Sal...	219	5e-60	30%
WP_102547170.1 group II intron reverse transcriptase/maturase ...	218	5e-60	32%
WP_102458767.1 MULTISPECIES: group II intron reverse transcrip...	217	6e-60	32%
ECF1699849.1 group II intron reverse transcriptase/maturase [S...	219	6e-60	30%
WP_086113864.1 group II intron reverse transcriptase/maturase ...	219	6e-60	30%
WP_136890147.1 group II intron reverse transcriptase/maturase ...	219	7e-60	31%
WP_047885252.1 group II intron reverse transcriptase/maturase ...	219	7e-60	31%
EAB5051756.1 group II intron reverse transcriptase/maturase [S...	219	7e-60	30%
EBY6522539.1 group II intron reverse transcriptase/maturase [S...	219	7e-60	30%
PMI99193.1 group II intron reverse transcriptase/maturase [Vib...	217	7e-60	32%
WP_107301457.1 group II intron reverse transcriptase/maturase ...	219	8e-60	31%
ECC9828226.1 group II intron reverse transcriptase/maturase [S...	218	8e-60	30%
WP_136994725.1 group II intron reverse transcriptase/maturase ...	217	8e-60	32%
OJY90761.1 group II intron reverse transcriptase/maturase [Xan...	218	9e-60	30%
WP_102504492.1 group II intron reverse transcriptase/maturase ...	217	9e-60	32%
WP_102570800.1 group II intron reverse transcriptase/maturase ...	217	9e-60	32%
EAA8323493.1 group II intron reverse transcriptase/maturase [S...	218	1e-59	30%
WP_148523617.1 group II intron reverse transcriptase/maturase ...	218	1e-59	30%
WP_148505600.1 group II intron reverse transcriptase/maturase ...	218	1e-59	30%
WP_136889514.1 group II intron reverse transcriptase/maturase ...	218	1e-59	30%
WP_102483506.1 group II intron reverse transcriptase/maturase ...	216	1e-59	32%
AKN39171.1 Retron-type RNA-directed DNA polymerase [Vibrio spl...	218	1e-59	32%
WP_100874669.1 group II intron reverse transcriptase/maturase ...	218	2e-59	31%
WP_136887894.1 group II intron reverse transcriptase/maturase ...	218	2e-59	30%
WP_005397527.1 MULTISPECIES: group II intron reverse transcrip...	217	2e-59	30%
CCN47380.1 Retron-type reverse transcriptase [Vibrio nigripulc...	218	2e-59	30%
WP_068144101.1 group II intron reverse transcriptase/maturase ...	218	2e-59	30%
KFD97154.1 HNH endonuclease family protein [Vibrio cholerae]	217	3e-59	30%
WP_144361825.1 group II intron reverse transcriptase/maturase ...	217	3e-59	31%
WP_038884138.1 group II intron reverse transcriptase/maturase ...	217	3e-59	31%
EMP83513.1 HNH endonuclease family protein [Vibrio cholerae O1...	217	3e-59	30%
EBW3237531.1 group II intron reverse transcriptase/maturase [S...	217	3e-59	31%
EAA4369619.1 group II intron reverse transcriptase/maturase [S...	217	3e-59	30%
ECC9153659.1 group II intron reverse transcriptase/maturase [S...	217	3e-59	30%
KXS52135.1 RNA-directed DNA polymerase [Marinobacter sp. T13-3]	218	3e-59	31%
WP_132970156.1 group II intron reverse transcriptase/maturase ...	215	4e-59	32%
WP_102776167.1 MULTISPECIES: group II intron reverse transcrip...	217	4e-59	31%
WP_069216972.1 group II intron reverse transcriptase/maturase ...	217	4e-59	30%

############

Query= ORF19

Length=265

Sequences producing significant alignments:	Score (Bits)	E Value	Max Ident
AYO45675.1 hypothetical protein [Caulerpa cupressoides]	295	3e-98	71%
YP_009326793.1 hypothetical protein [Caulerpa racemosa]	256	9e-82	53%
YP_009519137.1 hypothetical protein [Caulerpa verticillata]	241	6e-78	94%
YP_009519138.1 hypothetical protein [Caulerpa verticillata]	182	8e-55	84%
YP_009519483.1 hypothetical protein [Rhipiliopsis peltata]	183	2e-54	49%
YP_009519202.1 hypothetical protein [Caulerpa verticillata]	140	3e-38	64%
ASY96129.1 hypothetical protein [Tetradesmus obliquus]	99.0	8e-21	34%

############

Query= ORF21

Length=153

Sequences producing significant alignments:	Score (Bits)	E Value	Max Ident
YP_009472793.1 hypothetical protein [Caulerpa manorensis]	259	6e-86	95%
YP_009306232.1 hypothetical protein [Derbesia sp. WEST4838]	153	2e-44	56%
ALO20956.1 putative HNH homing endonuclease [Microglena monadina]	99.8	2e-23	39%
ANQ46363.1 hypothetical protein [Tetrabaena socialis]	101	3e-23	38%
YP_009184809.1 putative HNH homing endonuclease [Jenufa perfor...	94.4	1e-20	40%
AWI68513.1 putative HNH homing endonuclease [Pediastrum duplex]	92.0	1e-19	37%
ATW62840.1 homing endonuclease [Synechococcus phage S-CBWM1]	77.8	2e-14	37%
YP_636211.1 putative site-specific DNA endonuclease [Tupiella ...	73.6	6e-13	31%
YP_635978.1 putative site-specific DNA endonuclease [Tetradesm...	71.2	9e-12	40%
YP_008770907.1 HNH homing endonuclease [Salmonella phage Maynard]	66.6	2e-10	56%
ALO20952.1 putative HNH homing endonuclease [Microglena monadina]	66.6	4e-10	32%
AWX53465.1 hypothetical protein [Scotinosphaera sp. NIES-154]	66.2	6e-10	38%
YP_009104998.1 putative HNH homing endonuclease [Watanabea ren...	62.4	3e-09	37%
YP_009491939.1 putative site-specific DNA endonuclease [Pedias...	64.3	3e-09	45%
YP_764437.1 putative site-specific DNA endonuclease [Stigeoclo...	63.9	5e-09	50%
YP_009367897.1 putative HNH homing endonuclease [Hazenia capsu...	63.5	7e-09	36%
ALO21403.1 putative HNH homing endonuclease [Golenkinia longis...	62.8	8e-09	35%
AIT94640.1 putative HNH homing endonuclease [Chlorosarcina bre...	60.1	1e-08	36%
AYN55696.1 HNH homing endonuclease [Dickeya phage Kamild]	62.0	1e-08	54%
AOY36013.1 putative HNH homing endonuclease [Hariotina sp. MMO...	62.4	2e-08	33%
YP_009364130.1 putative HNH homing endonuclease [Hydrodictyon ...	60.8	5e-08	32%
ALO20951.1 putative HNH homing endonuclease [Microglena monadina]	61.2	6e-08	31%
ALO63172.1 putative HNH homing endonuclease [Chloromonas perfo...	61.2	6e-08	35%
YP_005089834.1 orf294 gene product [Dunaliella salina]	60.5	1e-07	36%
YP_009629468.1 HNH homing endonuclease [Coelastrella saipanensis]	59.7	1e-07	41%
YP_002000418.1 putative HNH homing endonuclease [Oedogonium ca...	59.7	2e-07	31%
YP_005089835.1 orf222 gene product [Dunaliella salina]	58.9	2e-07	37%
QAU05129.1 putative homing endonuclease [Pseudomonas phage Henu5]	57.8	2e-07	26%
ASY96103.1 hypothetical protein [Tetradesmus obliquus]	59.3	3e-07	29%
RYE21316.1 HNH endonuclease [Sphingobacteriaceae bacterium]	57.0	5e-07	31%
AWX53130.1 hypothetical protein [Blidingia minima]	57.8	6e-07	32%
YP_009629473.1 putative HNH homing endonuclease [Coelastrella ...	55.8	9e-07	33%
YP_002000413.1 putative HNH homing endonuclease [Oedogonium ca...	55.8	1e-06	36%
AUR97715.1 nuclease associated modular domain 3 protein [Vibri...	56.6	2e-06	42%
YP_004009436.1 putative HNH family homing endonuclease [Acinet...	54.7	4e-06	29%
YP_002922087.1 Homing endonuclease [Escherichia phage JSE]	55.5	7e-06	44%
YP_009280092.1 hypothetical protein MP1_gp0234 [Morganella pha...	54.3	1e-05	31%
AWI68321.1 putative HNH homing endonuclease [Pediastrum duplex]	53.1	2e-05	33%
AUV63985.1 HNH homing endonuclease [Shigella phage Sf20]	53.1	3e-05	28%
AUE22559.1 homing endonuclease [Aeromonas phage Ah1]	53.1	3e-05	43%
YP_007004261.1 homing endonuclease [Enterobacteria phage Bp7]	53.1	3e-05	22%
MBV36246.1 hypothetical protein [Rickettsiales bacterium]	53.1	3e-05	42%
YP_007890152.1 putative site-specific DNA endonuclease [Pleodo...	52.8	4e-05	31%
YP_003358572.1 putative homing endonuclease [Shigella phage Ag3]	52.4	5e-05	32%
AXF53330.1 hypothetical protein [Escherichia virus KFS-EC]	52.0	7e-05	29%
ADJ39722.1 homing endonuclease [Enterobacteria phage T4T]	52.4	7e-05	31%
YP_009030888.1 homing endonuclease [Escherichia phage vB_EcoM_...	52.4	7e-05	31%
YP_009148455.1 putative homing endonuclease [Escherichia phage...	52.4	8e-05	31%
YP_009284187.1 putative homing endonuclease [Escherichia phage...	52.4	8e-05	31%
YP_007501262.1 homing endonuclease [Salmonella phage vB_SenMS16]	51.6	1e-04	41%
YP_002117666.1 homing endonuclease [Rhizobium phage 16-3]	51.6	1e-04	39%
YP_004581389.1 orf265 gene product [Schizomeris leibleinii]	51.6	1e-04	42%
WP_148098846.1 HNH endonuclease [Enterobacter hormaechei]	49.3	1e-04	36%
QBO64613.1 putative HNH endonuclease [Escherichia phage vB_Eco...	52.0	1e-04	36%
YP_004300648.1 putative Mob-like homing endonuclease HNH famil...	50.8	1e-04	27%
NP_049674.1 MobB homing endonuclease [Escherichia virus T4]	51.6	1e-04	30%
YP_007890150.1 putative site-specific DNA endonuclease [Pleodo...	51.6	1e-04	48%
QBQ79945.1 group I intron endonuclease [Escherichia phage vB_E...	50.8	2e-04	30%
AUS02050.1 HNH nuclease [Vibrio phage 2.058.O._10N.286.46.B8]	50.8	2e-04	36%
YP_008060581.1 putative MobE-like endonuclease [Escherichia ph...	50.8	2e-04	54%
AEI71212.1 HNH endonuclease [EBPR siphovirus 1]	50.4	2e-04	40%
AON97067.1 putative Mob-like HNH homing endonuclease [Cronobac...	50.8	2e-04	54%
AUS02782.1 nuclease associated modular domain 3 protein [Vibri...	50.8	2e-04	38%
AOG16184.1 putative Mob-like HNH homing endonuclease [Cronobac...	50.4	2e-04	54%
YP_007674438.1 hypothetical protein SWZG_00073 [Synechococcus ...	49.3	3e-04	38%
YP_009615704.1 MobB-like homing endonuclease [Salmonella phage...	50.1	4e-04	42%
YP_006986332.1 putative Mob-like HNH homing endonuclease [Cron...	49.7	4e-04	54%
SCN45924.1 Putative homing endonuclease [Cronobacter phage Pet...	49.3	5e-04	34%
ARK07836.1 putative homing endonuclease [Aeromonas phage phiA8...	50.1	5e-04	54%
RHH45605.1 HNH endonuclease [Citrobacter portucalensis]	49.3	8e-04	41%
WP_147355674.1 HNH endonuclease [Citrobacter portucalensis]	49.3	8e-04	41%
AYJ74548.1 homing endonuclease [Yersinia phage PYPS2T]	48.9	8e-04	35%
P09753.1 RecName: Full=Uncharacterized 38.5 kDa protein in psb...	49.3	9e-04	44%
BAR28234.1 hypothetical protein [uncultured Mediterranean phag...	48.9	9e-04	40%
YP_005089832.1 orf192 gene product [Dunaliella salina]	48.5	9e-04	29%
QCW23038.1 hypothetical protein [Synechococcus phage S-B05]	47.8	0.001	45%
AVO22872.1 putative homing endonuclease [Erwinia phage vB_EamM...	48.9	0.001	39%

```
ALO63248.1 putative HNH homing endonuclease [Chlamydomonas app...  49.3    0.001  44%
NP_049844.1 MobE homing endonuclease [Escherichia virus T4]        47.4    0.001  37%
YP_009618315.1 hypothetical protein Cf1_00266 [Citrobacter pha...  48.5    0.001  47%
ADJ39953.1 homing endonuclease [Enterobacteria phage T4T]          48.1    0.002  37%
VEV88876.1 Phage-associated homing endonuclease [Yersinia phag...  48.1    0.002  40%
YP_008531965.1 putative homing endonuclease [Klebsiella virus ...  48.1    0.002  36%
YP_003858492.1 putative Mob-like HNH homing endonuclease [Esch...  48.1    0.002  36%
AWD91901.1 homing endonuclease [Enterobacteria phage vB_EcoM_I...  47.8    0.002  40%
ASZ77353.1 homing endonuclease [Escherichia phage ECO4]            47.8    0.002  36%
ARK07878.1 putative homing endonuclease [Aeromonas phage phiA8...  48.1    0.002  51%
QBO64906.1 hypothetical protein G50_00032 [Escherichia phage v...  47.8    0.002  40%
YP_009288835.1 putative HNH homing endonuclease [Klebsiella ph...  47.8    0.003  30%
WP_133330109.1 HNH endonuclease [Oxalobacteraceae bacterium SA...  46.6    0.003  44%
YP_009102983.1 hypothetical protein DA66_0144 [Dickeya phage R...  47.8    0.003  33%
AUG88058.1 homing endonuclease [Klebsiella phage May]              47.4    0.003  35%
AUS02988.1 HNH nuclease [Vibrio phage 2.275.O._10N.286.54.E11]     47.8    0.003  45%
YP_009168793.1 putative homing endonuclease [Escherichia phage...  47.8    0.003  35%
YP_802996.1 putative site-specific intron-like DNA endonucleas...  47.4    0.003  39%
NP_943926.1 MobE homing endonuclease [Aeromonas virus Aeh1]        47.4    0.003  41%
YP_009210148.1 Homing endonuclease [Enterobacteria phage JSE] ...  47.4    0.003  39%
ANZ50908.1 homing endonuclease [Enterobacteria phage Aplg8]        47.4    0.003  39%
AUM58396.1 putative homing endonuclease [Proteus phage phiP4-3]    47.4    0.003  36%
WP_080181637.1 HNH endonuclease [Salmonella enterica]              47.4    0.003  39%

  Database: All non-redundant GenBank CDS translations+PDB+SwissProt+PIR+PRF
excluding environmental samples from WGS projects
    Posted date:  Aug 30, 2019  12:43 AM
  Number of letters in database: 84,697,594,569
  Number of sequences in database:  234,979,574

Lambda      K        H
   0.320    0.137    0.401
Gapped
Lambda      K        H
   0.267   0.0410    0.140
Matrix: BLOSUM62
Gap Penalties: Existence: 11, Extension: 1
Number of Sequences: 234979574
Number of Hits to DB: 90961860
Number of extensions: 67340
Number of successful extensions: 15796
Number of sequences better than 100: 848
Number of HSP's better than 100 without gapping: 0
Number of HSP's gapped: 14739
Number of HSP's successfully gapped: 1416
Length of database: 84697594569
T: 21
A: 40
X1: 16 (7.4 bits)
X2: 38 (14.6 bits)
X3: 64 (24.7 bits)
S1: 41 (20.4 bits)
ka-blk-alpha gapped: 1.9
ka-blk-alpha ungapped: 0.7916
ka-blk-alpha_v gapped: 42.6028
ka-blk-alpha_v ungapped: 4.96466
ka-blk-sigma gapped: 43.6362
```
