# Supplementary material for: Promising prospects of nanopore sequencing for algal hologenomics and structural variation discovery
Source: BMC Genomics. 2019 Nov 13;20:850. doi: 10.1186/s12864-019-6248-2 (PMC6854639; doi:10.1186/s12864-019-6248-2)
Supplement: Supplementary file 17 — Additional file 17: Figure S15. BLASTp report for ORF7, ORF8, and ORF9. [file 12864_2019_6248_MOESM17_ESM.pdf]

BLASTP 2.10.0+
Reference: Stephen F. Altschul, Thomas L. Madden, Alejandro A. Schaffer, Jinghui Zhang, Zheng Zhang, Webb Miller, and David J. Lipman (1997), "Gapped BLAST and PSI-BLAST: a new generation of protein database search programs", Nucleic Acids Res. 25:3389-3402.

Reference for compositional score matrix adjustment: Stephen F. Altschul, John C. Wootton, E. Michael Gertz, Richa Agarwala, Aleksandr Morgulis, Alejandro A. Schaffer, and Yi-Kuo Yu (2005) "Protein database searches using compositionally adjusted substitution matrices", FEBS J. 272:5101-5109.

RID: SA820JPM014

Database: All non-redundant GenBank CDS translations+PDB+SwissProt+PIR+PRF excluding environmental samples from WGS projects
234,979,574 sequences; 84,697,594,569 total letters

############

Query= ORF7

Length=376

| Sequences producing significant alignments: | Score (Bits) | E Value | Max Ident |
|---|---|---|---|
| YP_009519162.1 hypothetical protein [Caulerpa verticillata] | 335 | 3e-111 | 61% |
| YP_009472820.1 hypothetical protein [Caulerpa manorensis] | 259 | 1e-80 | 42% |
| YP_009519448.1 hypothetical protein [Rhipiliopsis peltata] | 206 | 1e-59 | 56% |
| AYC64487.1 hypothetical protein [Pseudochlorodesmis sp. HV01306c] | 197 | 9e-58 | 54% |
| AOP19087.1 hypothetical protein [Halimeda discoidea] | 184 | 2e-53 | 58% |
| YP_009306362.1 hypothetical protein [Caulerpa cliftonii] | 106 | 4e-21 | 31% |
| YP_009519140.1 hypothetical protein [Caulerpa verticillata] | 97.4 | 8e-21 | 48% |
| ARR28454.1 hypothetical protein [Caulerpa okamurae] | 89.7 | 2e-17 | 34% |
| AYC64090.1 hypothetical protein [Johnson-sea-linkia profunda] | 89.7 | 1e-15 | 27% |
| AOP19034.1 hypothetical protein [Halimeda discoidea] | 89.4 | 2e-15 | 34% |
| YP_009472588.1 hypothetical protein [Rhipilia penicilloides] | 88.6 | 4e-15 | 28% |
| YP_009130586.1 putative DNA primase [Tydemania expeditionis] | 86.7 | 7e-15 | 29% |
| YP_009326773.1 hypothetical protein [Caulerpa racemosa] | 86.3 | 2e-14 | 28% |
| YP_009472859.1 hypothetical protein [Caulerpa manorensis] | 85.5 | 4e-14 | 28% |
| YP_009518867.1 hypothetical protein [Boodleopsis pusilla] | 83.2 | 2e-13 | 31% |
| WP_106459521.1 DUF3854 domain-containing protein [Aphanothece ... | 74.3 | 2e-10 | 30% |
| OQX20052.1 hypothetical protein BWK76_02530 [Desulfobulbaceae ... | 73.2 | 4e-10 | 29% |
| WP_106459359.1 DUF3854 domain-containing protein [Aphanothece ... | 72.0 | 1e-09 | 29% |
| WP_106872064.1 MULTISPECIES: hypothetical protein [unclassifie... | 60.5 | 5e-06 | 28% |
| WP_048670661.1 toprim domain-containing protein [Candidatus Co... | 57.4 | 5e-05 | 29% |
| WP_117948860.1 hypothetical protein [Faecalibacterium prausnit... | 56.6 | 8e-05 | 27% |
| ASC70311.1 hypothetical protein XM38_012480 [Halomicronema hon... | 55.1 | 2e-04 | 27% |
| WP_137455024.1 hypothetical protein [Halomicronema hongdechloris] | 55.1 | 3e-04 | 27% |
| WP_005329991.1 phage/plasmid primase P4 family domain [Coryneb... | 53.9 | 5e-04 | 24% |
| WP_080099994.1 hypothetical protein [Vibrio cholerae] | 53.9 | 6e-04 | 26% |
| EKY32300.1 hypothetical protein OSU_2061 [Vibrio cholerae PS15] | 53.5 | 8e-04 | 26% |
| WP_015537438.1 phage/plasmid primase, P4 family, C-terminal do... | 52.8 | 0.001 | 25% |
| WP_097783161.1 hypothetical protein [Faecalibacterium prausnit... | 52.4 | 0.002 | 25% |
| CVI73287.1 hypothetical protein NDGK_02952 [Clostridiales bact... | 51.2 | 0.002 | 25% |
| HBJ06247.1 TPA: hypothetical protein [Erysipelotrichaceae bact... | 50.8 | 0.004 | 29% |

| | | | |
|---|---|---|---|
| TAD79303.1 hypothetical protein EA001_04585 [Oscillatoriales c... | 51.2 | 0.004 | 27% |
| YP_009519502.1 hypothetical protein [Rhipiliopsis peltata] | 47.4 | 0.005 | 33% |
| PXX83167.1 hypothetical protein DLY76_13175 [Staphylococcus wa... | 50.8 | 0.005 | 27% |
| WP_089826771.1 DNA primase [Clostridiales bacterium CHKCI001] | 50.1 | 0.006 | 26% |
| WP_122135129.1 hypothetical protein [Staphylococcus warneri] | 50.4 | 0.006 | 27% |
| WP_058709749.1 hypothetical protein [Staphylococcus warneri] | 50.4 | 0.007 | 27% |
| WP_117726019.1 hypothetical protein [Staphylococcus warneri] | 50.4 | 0.007 | 27% |
| WP_099533992.1 DUF3854 domain-containing protein [Limnothrix s... | 50.4 | 0.008 | 25% |
| WP_122135133.1 hypothetical protein [Staphylococcus warneri] | 50.4 | 0.008 | 27% |
| OCQ94853.1 hypothetical protein BCR12_14730 [Limnothrix sp. P1... | 50.4 | 0.008 | 25% |
| YP_009519361.1 hypothetical protein [Pseudocodium devriesii] | 48.5 | 0.009 | 32% |
| WP_051669969.1 hypothetical protein [Methylomonas sp. LW13] | 49.7 | 0.011 | 27% |
| WP_050044965.1 DUF3854 domain-containing protein [Tolypothrix ... | 49.7 | 0.014 | 25% |
| BAY87916.1 primase P4 [Calothrix parasitica NIES-267] | 49.7 | 0.015 | 24% |
| WP_087545683.1 hypothetical protein [Nostocales cyanobacterium... | 49.3 | 0.015 | 27% |
| WP_096661640.1 DUF3854 domain-containing protein [Calothrix pa... | 49.7 | 0.016 | 24% |
| WP_134356385.1 toprim domain-containing protein [Nitrosococcus... | 48.9 | 0.021 | 26% |
| WP_114735354.1 hypothetical protein [Vibrio cholerae] | 48.9 | 0.025 | 24% |
| WP_148519512.1 hypothetical protein [Vibrio cholerae] | 48.5 | 0.029 | 24% |
| WP_082755228.1 hypothetical protein [Bradyrhizobium diazoeffic... | 48.5 | 0.032 | 27% |
| YP_009519210.1 hypothetical protein [Caulerpa verticillata] | 48.1 | 0.036 | 36% |
| ALO50062.1 E1 protein [Deltapapillomavirus 5] | 47.8 | 0.046 | 29% |
| WP_081712488.1 hypothetical protein [Gordonia amicalis] | 47.4 | 0.060 | 30% |
| WP_076343410.1 hypothetical protein [Paludisphaera borealis] | 47.4 | 0.079 | 26% |
| WP_143052189.1 hypothetical protein [Halobacterium jilantaiense] | 47.0 | 0.090 | 26% |
| BBD63650.1 hypothetical protein NIES2109_65250 [Nostoc sp. HK-01] | 47.0 | 0.10 | 27% |
| YP_009519427.1 hypothetical protein [Rhipiliopsis peltata] | 46.2 | 0.12 | 38% |
| WP_015014502.1 phage/plasmid primase [Acidovorax sp. KKS102] | 45.4 | 0.27 | 27% |
| WP_069652414.1 toprim domain-containing protein [Aeromonas cav... | 45.4 | 0.28 | 26% |
| TMQ25139.1 NTP-binding protein [Deltaproteobacteria bacterium] | 45.1 | 0.32 | 27% |
| WP_099869324.1 NTP-binding protein [Mycobacterium heckeshornense] | 44.7 | 0.44 | 27% |
| WP_071700079.1 hypothetical protein [Mycobacterium heckeshorne... | 44.7 | 0.49 | 27% |
| WP_055185464.1 hypothetical protein [Faecalibacterium prausnit... | 43.9 | 0.79 | 27% |
| WP_117554111.1 hypothetical protein [Faecalibacterium prausnit... | 43.9 | 0.81 | 27% |
| WP_120020883.1 hypothetical protein [Faecalibacterium sp. AF10... | 43.9 | 0.85 | 23% |
| WP_080793152.1 hypothetical protein [Corynebacterium sp. Marse... | 43.5 | 0.93 | 21% |
| AVP41438.1 DNA primase [Streptomyces phage Yara] | 43.9 | 0.95 | 28% |
| WP_146372635.1 DUF3854 domain-containing protein [Planctomycet... | 43.5 | 1.0 | 25% |
| AXQ00533.1 E1 protein [Okapia johnstoni papillomavirus 1] | 43.5 | 1.1 | 28% |
| WP_103918394.1 hypothetical protein [Thiotrichales bacterium H... | 43.5 | 1.1 | 25% |
| WP_127066654.1 hypothetical protein [Cellulosilyticum sp. WCF-2] | 43.1 | 1.1 | 25% |
| WP_025988927.1 hypothetical protein [Mycobacteroides abscessus] | 43.5 | 1.2 | 27% |
| SHP30503.1 phage/plasmid primase, P4 family, C-terminal domain... | 43.5 | 1.2 | 27% |
| WP_145718318.1 hypothetical protein [Mesorhizobium tianshanense] | 43.5 | 1.2 | 30% |
| WP_074243874.1 unnamed protein product | 43.5 | 1.2 | 27% |
| WP_052617223.1 hypothetical protein [Mycobacteroides abscessus] | 43.5 | 1.2 | 26% |
| CPS32393.1 phage/plasmid primase%2C P4 family%2C C-terminal do... | 43.5 | 1.3 | 27% |
| QEA05367.1 hypothetical protein KBTEX_01688 [uncultured organism] | 43.1 | 1.3 | 25% |
| CPU37683.1 bacteriophage protein [Mycobacteroides abscessus] | 43.5 | 1.3 | 27% |
| WP_052536874.1 hypothetical protein [Mycobacteroides abscessus] | 43.5 | 1.3 | 27% |
| WP_074269985.1 hypothetical protein [Mycobacteroides abscessus] | 43.1 | 1.4 | 27% |
| WP_100451585.1 hypothetical protein [Mycobacteroides abscessus] | 43.1 | 1.4 | 27% |
| WP_100460742.1 hypothetical protein [Mycobacteroides abscessus] | 43.1 | 1.5 | 24% |
| WP_119570835.1 hypothetical protein [Mycobacteroides abscessus] | 43.1 | 1.5 | 27% |
| OGS01264.1 hypothetical protein A3G41_05925 [Elusimicrobia bac... | 43.1 | 1.6 | 21% |
| SHP11590.1 phage/plasmid primase [Mycobacteroides abscessus su... | 43.1 | 1.7 | 27% |
| YP_009508736.1 E1 protein [Giraffa camelopardalis papillomavir... | 42.7 | 1.7 | 28% |
| SHP06125.1 bacteriophage protein [Mycobacteroides abscessus su... | 42.4 | 2.6 | 24% |
| WP_144681351.1 hypothetical protein [Desulfobotulus alkaliphilus] | 42.4 | 2.6 | 30% |
| WP_074232794.1 hypothetical protein [Mycobacteroides abscessus] | 42.4 | 2.8 | 24% |
| WP_098923763.1 hypothetical protein [Faecalibacterium prausnit... | 42.0 | 2.9 | 23% |
| SKI81432.1 bacteriophage protein [Mycobacteroides abscessus su... | 42.4 | 3.0 | 24% |
| WP_064996905.1 hypothetical protein [Mycolicibacter heraklione... | 42.0 | 3.6 | 26% |
| PKM60256.1 DNA primase [Firmicutes bacterium HGW-Firmicutes-4] | 42.0 | 3.9 | 25% |

| | | | |
|---|---|---|---|
| WP_006347292.1 NTP-binding protein [Streptomyces tsukubensis] | 41.2 | 4.8 | 25% |
| PZV21913.1 DNA primase [Snowella sp.] | 41.6 | 5.1 | 28% |
| KUL51672.1 NTP-binding protein [Streptomyces sp. NRRL S-1521] | 41.2 | 5.3 | 26% |
| KFG75654.1 hypothetical protein FM21_05885 [Streptomyces luteus] | 41.2 | 5.4 | 26% |
| WP_107063888.1 NTP-binding protein [Streptomyces luteus] | 41.2 | 5.6 | 26% |
| ALL29332.1 E1 [Bovine papillomavirus type 14] | 41.2 | 5.7 | 29% |

############

Query= ORF8

Length=660

| Sequences producing significant alignments: | Score (Bits) | E Value | Max Ident |
|---|---|---|---|
| YP_009326860.1 hypothetical protein [Caulerpa racemosa] | 253 | 1e-74 | 43% |
| AYO45732.1 hypothetical protein [Caulerpa cupressoides] | 182 | 3e-49 | 40% |
| YP_009519211.1 hypothetical protein [Caulerpa verticillata] | 176 | 4e-43 | 30% |
| YP_009519359.1 hypothetical protein [Pseudocodium devriesii] | 152 | 3e-36 | 29% |
| YP_009519163.1 hypothetical protein [Caulerpa verticillata] | 136 | 4e-34 | 62% |
| YP_009326773.1 hypothetical protein [Caulerpa racemosa] | 151 | 4e-34 | 26% |
| YP_009519209.1 hypothetical protein [Caulerpa verticillata] | 137 | 1e-33 | 52% |
| YP_009472855.1 hypothetical protein [Caulerpa manorensis] | 147 | 5e-33 | 26% |
| AYC64085.1 hypothetical protein [Johnson-sea-linkia profunda] | 145 | 6e-33 | 25% |
| WP_008287183.1 bifunctional 3'-5' exonuclease/DNA polymerase [... | 136 | 6e-30 | 26% |
| WP_121011606.1 bifunctional 3'-5' exonuclease/DNA polymerase [... | 132 | 1e-28 | 26% |
| WP_121922680.1 bifunctional 3'-5' exonuclease/DNA polymerase [... | 129 | 1e-27 | 30% |
| WP_028949995.1 bifunctional 3'-5' exonuclease/DNA polymerase [... | 128 | 2e-27 | 26% |
| EGS31161.1 DNA-directed DNA polymerase [Peptoniphilus sp. oral... | 126 | 3e-27 | 27% |
| WP_012673655.1 bifunctional 3'-5' exonuclease/DNA polymerase [... | 127 | 5e-27 | 26% |
| MAV17669.1 DNA polymerase I [Gammaproteobacteria bacterium] | 127 | 1e-26 | 31% |
| WP_072469393.1 DNA polymerase I [Urinacoccus massiliensis] | 125 | 6e-26 | 27% |
| WP_034440597.1 DNA polymerase I [Tissierellia bacterium S5-A11] | 125 | 7e-26 | 27% |
| WP_097041874.1 DNA polymerase I [Terribacillus aidingensis] | 125 | 1e-25 | 26% |
| WP_099208946.1 DNA polymerase I [Urinacoccus sp. Marseille-P3926] | 124 | 1e-25 | 27% |
| WP_068868882.1 DNA polymerase I [Planococcus plakortidis] | 124 | 1e-25 | 28% |
| AKE21663.1 DNA polymerase A family protein [Francisella tulare... | 118 | 2e-25 | 30% |
| WP_131748623.1 DNA polymerase I [Anaerococcus prevotii] | 124 | 2e-25 | 27% |
| WP_095220162.1 DNA polymerase I [Terribacillus saccharophilus] | 124 | 2e-25 | 26% |
| WP_095272594.1 DNA polymerase I [Terribacillus saccharophilus] | 124 | 2e-25 | 26% |
| RUM46834.1 bifunctional 3'-5' exonuclease/DNA polymerase [Hydr... | 122 | 2e-25 | 26% |
| AYC64023.1 hypothetical protein [Halimeda micronesica] | 112 | 2e-25 | 45% |
| WP_095232974.1 DNA polymerase I [Terribacillus saccharophilus] | 123 | 2e-25 | 26% |
| WP_095260824.1 DNA polymerase I [Terribacillus saccharophilus] | 123 | 3e-25 | 26% |
| WP_095226616.1 DNA polymerase I [Terribacillus saccharophilus] | 123 | 3e-25 | 26% |
| WP_095222178.1 DNA polymerase I [Virgibacillus sp. 7505] | 123 | 3e-25 | 26% |
| WP_077308099.1 DNA polymerase I [Terribacillus halophilus] | 123 | 4e-25 | 26% |
| WP_142330819.1 DNA polymerase I [Bacillus thuringiensis] | 122 | 4e-25 | 26% |
| WP_121300248.1 DNA polymerase I [Planococcus citreus] | 122 | 5e-25 | 28% |
| WP_069718151.1 DNA polymerase I [Bacillus solimangrovi] | 122 | 6e-25 | 26% |
| PFP11927.1 DNA polymerase I [Bacillus thuringiensis] | 122 | 6e-25 | 26% |
| PMP76048.1 bifunctional 3'-5' exonuclease/DNA polymerase [Sulf... | 121 | 6e-25 | 24% |
| PFE06921.1 DNA polymerase I [Bacillus cereus] | 121 | 1e-24 | 26% |
| OON59049.1 DNA polymerase I [Klebsiella pneumoniae] | 118 | 1e-24 | 26% |
| WP_040955141.1 DNA polymerase I [Virgibacillus sp. SK37] | 121 | 1e-24 | 24% |
| WP_141532717.1 DNA polymerase I [Bacillus cereus] | 121 | 1e-24 | 26% |
| WP_093879404.1 DNA polymerase I [Terribacillus saccharophilus] | 121 | 1e-24 | 25% |
| TKJ03686.1 DNA polymerase I [Bacillus cereus] | 119 | 1e-24 | 26% |
| WP_141525460.1 DNA polymerase I [Bacillus cereus] | 121 | 1e-24 | 26% |
| WP_140386899.1 MULTISPECIES: DNA polymerase I [Bacillus cereus... | 121 | 1e-24 | 26% |
| PFL69735.1 DNA polymerase I [Bacillus cereus] | 121 | 1e-24 | 26% |
| PFB29359.1 DNA polymerase I [Bacillus cereus] | 121 | 1e-24 | 26% |

| | | | |
|---|---|---|---|
| PMP60411.1 bifunctional 3'-5' exonuclease/DNA polymerase [Sulf... | 120 | 2e-24 | 24% |
| WP_141558566.1 DNA polymerase I [Bacillus thuringiensis] | 121 | 2e-24 | 26% |
| WP_142947063.1 DNA polymerase I [Bacillus cereus] | 120 | 2e-24 | 26% |
| WP_141538298.1 DNA polymerase I [Bacillus thuringiensis] | 120 | 2e-24 | 26% |
| PGW45597.1 DNA polymerase I [Bacillus thuringiensis] | 120 | 2e-24 | 26% |
| AHA74189.1 DNA polymerase I [Bacillus thuringiensis YBT-1518] | 120 | 2e-24 | 26% |
| WP_025117755.1 DNA polymerase I [Bacillus sp. H1m] | 120 | 2e-24 | 26% |
| WP_140392562.1 DNA polymerase I [Bacillus cereus] | 120 | 2e-24 | 26% |
| WP_080672436.1 DNA polymerase I [Bacillus thuringiensis] | 120 | 2e-24 | 26% |
| WP_075716589.1 MULTISPECIES: DNA polymerase I [Bacillus cereus... | 120 | 2e-24 | 26% |
| PEC73952.1 DNA polymerase I [Bacillus thuringiensis] | 120 | 2e-24 | 26% |
| WP_022853929.1 DNA polymerase I [Thermodesulfatator atlanticus] | 120 | 2e-24 | 28% |
| WP_053563570.1 MULTISPECIES: DNA polymerase I [Bacillus] | 120 | 2e-24 | 26% |
| WP_063548740.1 MULTISPECIES: DNA polymerase I [Bacillus cereus... | 120 | 2e-24 | 26% |
| TCL51840.1 DNA polymerase I [Thermolongibacillus altinsuensis] | 120 | 2e-24 | 25% |
| WP_000412829.1 MULTISPECIES: DNA polymerase I [Bacillus] | 120 | 2e-24 | 26% |
| WP_065229422.1 MULTISPECIES: DNA polymerase I [Bacillus cereus... | 120 | 2e-24 | 26% |
| PEE64345.1 DNA polymerase I [Bacillus thuringiensis] | 120 | 2e-24 | 26% |
| PES44316.1 DNA polymerase I [Bacillus thuringiensis] | 120 | 2e-24 | 26% |
| PEF03997.1 DNA polymerase I [Bacillus thuringiensis] | 120 | 2e-24 | 26% |
| WP_065485383.1 DNA polymerase I [Bacillus thuringiensis] | 120 | 2e-24 | 26% |
| AVP46514.1 DNA polymerase I [Bacillus cereus] | 120 | 2e-24 | 26% |
| WP_141505510.1 DNA polymerase I [Bacillus thuringiensis] | 120 | 2e-24 | 26% |
| WP_142327068.1 DNA polymerase I [Bacillus thuringiensis] | 120 | 2e-24 | 26% |
| PEO01438.1 DNA polymerase I [Bacillus cereus] | 120 | 2e-24 | 26% |
| WP_016512259.1 MULTISPECIES: DNA polymerase I [Bacillus] | 120 | 2e-24 | 26% |
| WP_141484265.1 DNA polymerase I [Bacillus thuringiensis] | 120 | 2e-24 | 26% |
| WP_078993673.1 DNA polymerase I [Bacillus thuringiensis] | 120 | 2e-24 | 26% |
| WP_141550417.1 DNA polymerase I [Bacillus thuringiensis] | 120 | 2e-24 | 26% |
| WP_113303084.1 DNA polymerase I [Bacillus cereus] | 120 | 2e-24 | 26% |
| WP_132947547.1 DNA polymerase I [Thermolongibacillus altinsuen... | 120 | 2e-24 | 25% |
| PEB73834.1 DNA polymerase I [Bacillus thuringiensis] | 120 | 2e-24 | 26% |
| WP_080469882.1 DNA polymerase I [Bacillus cereus] | 120 | 2e-24 | 26% |
| WP_145959744.1 DNA polymerase I [Bacillus cereus] | 120 | 2e-24 | 26% |
| WP_141493449.1 DNA polymerase I [Bacillus thuringiensis] | 120 | 2e-24 | 26% |
| COF45140.1 DNA polymerase I [Streptococcus pneumoniae] | 118 | 2e-24 | 26% |
| PFJ62672.1 DNA polymerase I [Bacillus thuringiensis] | 120 | 2e-24 | 26% |
| WP_080126339.1 DNA polymerase I [Bacillus cereus] | 120 | 3e-24 | 26% |
| WP_038562164.1 DNA polymerase I [Terribacillus goriensis] | 120 | 3e-24 | 25% |
| WP_080017654.1 MULTISPECIES: DNA polymerase I [Bacillus cereus... | 120 | 3e-24 | 26% |
| PEG03856.1 DNA polymerase I [Bacillus cereus] | 120 | 3e-24 | 26% |
| WP_142312045.1 DNA polymerase I [Bacillus cereus] | 120 | 3e-24 | 26% |
| PDX94771.1 DNA polymerase I [Bacillus thuringiensis] | 120 | 3e-24 | 26% |
| WP_144524277.1 DNA polymerase I [Bacillus thuringiensis] | 120 | 3e-24 | 26% |
| WP_060486537.1 DNA polymerase I [Bacillus wiedmannii] | 120 | 3e-24 | 26% |
| PED35322.1 DNA polymerase I [Bacillus cereus] | 120 | 3e-24 | 26% |
| WP_102707368.1 DNA polymerase I [Terribacillus goriensis] | 120 | 3e-24 | 25% |
| PHD63790.1 DNA polymerase I [Bacillus wiedmannii] | 120 | 3e-24 | 26% |
| WP_142399372.1 DNA polymerase I [Bacillus wiedmannii] | 120 | 3e-24 | 26% |
| WP_142309456.1 DNA polymerase I [Bacillus cereus] | 120 | 3e-24 | 26% |
| PRT05698.1 DNA polymerase I [Bacillus wiedmannii] | 120 | 3e-24 | 26% |
| WP_144463139.1 MULTISPECIES: DNA polymerase I [Bacillus] | 120 | 3e-24 | 25% |
| WP_120075084.1 DNA polymerase I [Bacillus fortis] | 120 | 3e-24 | 25% |

############

Query= ORF9

Length=651

| Sequences producing significant alignments: | Score (Bits) | E Value | Max Ident |
|---|---|---|---|

| | | | |
|---|---|---|---|
| AYO45733.1 hypothetical protein [Caulerpa cupressoides] | 760 | 0.0 | 65% |
| YP_009326774.1 hypothetical protein [Caulerpa racemosa] | 681 | 0.0 | 59% |
| YP_009472822.1 hypothetical protein [Caulerpa manorensis] | 662 | 0.0 | 64% |
| YP_009472858.1 hypothetical protein [Caulerpa manorensis] | 551 | 0.0 | 56% |
| ARR28462.1 hypothetical protein [Caulerpa okamurae] | 447 | 3e-150 | 70% |
| YP_009326795.1 hypothetical protein [Caulerpa racemosa] | 282 | 4e-86 | 59% |
| YP_009326809.1 hypothetical protein [Caulerpa racemosa] | 144 | 4e-35 | 68% |
| AYO45724.1 putative DNA polymerase I [Caulerpa cupressoides] | 88.6 | 1e-16 | 52% |
| YP_009472804.1 hypothetical protein [Caulerpa manorensis] | 77.4 | 2e-12 | 49% |
| AYC64086.1 hypothetical protein [Johnson-sea-linkia profunda] | 72.8 | 2e-09 | 25% |
| AYC64626.1 hypothetical protein [Halimeda minima] | 68.9 | 6e-09 | 29% |
| AYO45723.1 putative LAGLIDADG homing endonuclease [Caulerpa cu... | 60.1 | 6e-08 | 61% |
| YP_009519615.1 hypothetical protein [Udotea flabellum] | 67.4 | 7e-08 | 26% |
| ARR28439.1 hypothetical protein [Caulerpa okamurae] | 55.5 | 4e-05 | 43% |
| YP_009518872.1 hypothetical protein [Boodleopsis pusilla] | 55.1 | 4e-04 | 25% |
| AYC64617.1 hypothetical protein [Halimeda minima] | 54.3 | 7e-04 | 25% |

```
  Database: All non-redundant GenBank CDS translations+PDB+SwissProt+PIR+PRF
excluding environmental samples from WGS projects
    Posted date:  Aug 30, 2019  12:43 AM
  Number of letters in database: 84,697,594,569
  Number of sequences in database:  234,979,574

Lambda      K        H
   0.317    0.135    0.380
Gapped
Lambda      K        H
   0.267   0.0410    0.140
Matrix: BLOSUM62
Gap Penalties: Existence: 11, Extension: 1
Number of Sequences: 234979574
Number of Hits to DB: 90373741
Number of extensions: 155835
Number of successful extensions: 97294
Number of sequences better than 100: 4024
Number of HSP's better than 100 without gapping: 0
Number of HSP's gapped: 76902
Number of HSP's successfully gapped: 10950
Length of database: 84697594569
T: 21
A: 40
X1: 16 (7.3 bits)
X2: 38 (14.6 bits)
X3: 64 (24.7 bits)
S1: 41 (20.4 bits)
ka-blk-alpha gapped: 1.9
ka-blk-alpha ungapped: 0.7916
ka-blk-alpha_v gapped: 42.6028
ka-blk-alpha_v ungapped: 4.96466
ka-blk-sigma gapped: 43.6362
```
